# The genetic control of polyacetylenes involved in bitterness of carrots (*Daucus carota* L.): Identification of QTLs and candidate genes from the plant fatty acid metabolism

**DOI:** 10.1186/s12870-022-03484-1

**Published:** 2022-03-02

**Authors:** Frank Dunemann, Wanying He, Christoph Böttcher, Sven Reichardt, Thomas Nothnagel, Paul Heuvelmans, Freddy Hermans

**Affiliations:** 1 Julius Kühn-Institut (JKI), Institute for Breeding Research on Horticultural Crops, Erwin-Baur-Str. 27, 06484 Quedlinburg, Germany; 2 Julius Kühn-Institut (JKI), Institute for Ecological Chemistry, Plant Analysis and Stored Product Protection, Königin-Luise-Str. 19, 14195 Berlin, Germany; 3 Julius Kühn-Institut (JKI), Institute for Biosafety in Plant Biotechnology, Erwin-Baur-Str. 27, 06484 Quedlinburg, Germany; 4 Nunhems Netherlands BV, Napoleonsweg 152, 6083 AB Nunhem, The Netherlands

**Keywords:** *Daucus carota*, Polyacetylene, Falcarinol, Falcarindiol, HPLC/DAD, QTL mapping, Candidate gene, Fatty acid desaturase, *FAD2* gene family, *CER1 decarbonylase* gene

## Abstract

**Background:**

Falcarinol-type polyacetylenes (PAs) such as falcarinol (FaOH) and falcarindiol (FaDOH) are produced by several Apiaceae vegetables such as carrot, parsnip, celeriac and parsley. They are known for numerous biological functions and contribute to the undesirable bitter off-taste of carrots and their products. Despite their interesting biological functions, the genetic basis of their structural diversity and function is widely unknown. A better understanding of the genetics of the PA levels present in carrot roots might support breeding of carrot cultivars with tailored PA levels for food production or nutraceuticals.

**Results:**

A large carrot F_2_ progeny derived from a cross of a cultivated inbred line with an inbred line derived from a *Daucus carota* ssp*. commutatus* accession rich in PAs was used for linkage mapping and quantitative trait locus (QTL) analysis. Ten QTLs for FaOH and FaDOH levels in roots were identified in the carrot genome. Major QTLs for FaOH and FaDOH with high LOD values of up to 40 were identified on chromosomes 4 and 9. To discover putative candidate genes from the plant fatty acid metabolism, we examined an extended version of the inventory of the carrot *FATTY ACID DESATURASE2* (*FAD2*) gene family. Additionally, we used the carrot genome sequence for a first inventory of *ECERIFERUM1* (*CER1*) genes possibly involved in PA biosynthesis. We identified genomic regions on different carrot chromosomes around the found QTLs that contain several *FAD2* and *CER1* genes within their 2-LOD confidence intervals. With regard to the major QTLs on chromosome 9 three putative *CER1* decarbonylase gene models are proposed as candidate genes.

**Conclusion:**

The present study increases the current knowledge on the genetics of PA accumulation in carrot roots. Our finding that carrot candidate genes from the fatty acid metabolism are significantly associated with major QTLs for both major PAs, will facilitate future functional gene studies and a further dissection of the genetic factors controlling PA accumulation. Characterization of such candidate genes will have a positive impact on carrot breeding programs aimed at both lowering or increasing PA concentrations in carrot roots.

**Supplementary Information:**

The online version contains supplementary material available at 10.1186/s12870-022-03484-1.

## Background

Carrot is an economically important and world-wide grown root vegetable known as a multi-nutritional food source. The carrot root is one of the richest sources of provitamin A and contains several other bioactive natural products, which are recognized for their nutraceutical effects [1, 2]. Among the several chemical major classes the polyacetylenes (PAs) are a large group of phytochemicals, which are produced in higher plants of the families Apiaceae, Araliaceae and Asteraceae [3, 4]. Twelve different aliphatic C_17_-PAs have been characterized in carrots [4], and Busta et al. [5] recently added two further compounds to this list. The major PAs of carrot and other Apiaceae species are falcarindiol (FaDOH) and its presumed precursor falcarinol (FaOH), previously also known as carotatoxin [6]. In carrot root periderm FaDOH is the quantitatively predominating PA [5, 7] but the concentrations of FaOH and FaDOH depend on the carrot cultivar [8]. In addition, roots of some wild carrot relatives such as the subspecies *D. c. maximus, D. c. maritimus,* or *D. c. halophilus* were shown to have up to 20 times higher PA concentrations than roots of cultivated carrots [9, 10]. Next to other phytochemicals such as 6-methoxymellein, laserin and epilaserin the presence of higher amounts of C_17_-PAs has been shown to contribute to the undesirable bitter off-taste of carrots and their products [11–13]. The localization of PAs in exterior plant tissue layers such as the root periderm is also consistent with their assumed role in plant defense. Moreover, FaOH and FaDOH might action against some fungal carrot pathogens [14]. Accumulation of polyacetylenic phytoalexins, including FaOH, was observed in tomato fruits and leaves induced with *Cladosporium fulvum*, *Verticillium albo-atrum*, and *Fusarium oxysporum* [6]. In carrots, FaDOH could inhibit the in vitro development of the fungal leaf blight pathogen *Alternaria dauci,* and the levels increased in response to *A. dauci* inoculation [15]. With regard to putative effects of PAs on human health a wide range of biological activities have been reported for falcarinol-type PAs demonstrating that they exhibit potent anti-microbial, anti-inflammatory and anti-cancer effects [3, 4, 16–19].

Despite their interesting biological functions, the biosynthesis of PAs and its genetic basis are poorly understood. In higher plants PAs are produced from unsaturated fatty acids such as oleic acid and linoleic acid. It has been hypothesized that a diverse pathway from linolenic acid to unusual fatty acids, such as crepenynic and dehydrocrepenynic acid, is the major route for the biosynthesis of falcarinol-type PAs [5, 6]. The enzyme primarily responsible for the synthesis of linoleic acid from oleic acid is a Δ12-fatty acid desaturase [20]. Divergent forms of FATTY ACID DESATURASE 2  (FAD2) enzymes have diversified functionalities in fatty acid modification, e.g. catalyzing hydroxylation [21], epoxidation [22] and the formation of acetylenic bonds and conjugated double bonds [23]. The *Crep1* gene from *Crepis alpina* was the first cloned *FAD2* gene encoding a functional acetylenase. This enzyme accumulates large amounts of the acetylenic fatty acid crepenynic acid in the seeds of *C. alpina* [22].

*FAD2* is among the best-studied plant fatty acid desaturase gene families, in terms of both molecular and biochemical investigations. Since the cloning of a first plant *FAD2* gene in *A. thaliana* [24], *FAD2* genes have been isolated and characterized from many different plant species, including members of the *FAD2* gene family that possess diverse functional activities in fatty acid modification such as acetylenases. Only a single *FAD2* gene exists in *Arabidopsis*, but in most other plants multiple homologous *FAD2s* were found. Species with the largest known number of *FAD2s* are soybean (seven *FAD2* gene family members), cotton and tomato (9 genes), and safflower (11 genes) [25]. However, in the Apiaceae species *Petroselinum crispum* (parsley) 17 *FAD2* sequences were found [26], and in the carrot genome 24 *FAD2s* were previously reported [5]. This is the highest number of *FAD2* family members reported so far, indicating that functional divergence of *FAD2s* is not limited to oil seed crops [25].

The biochemical formation of FaDOH from its presumed precursor substance FaOH is largely unknown until now. Recently a pathogen-induced biosynthetic gene cluster was discovered in tomato and shown to be putatively involved in FaDOH production [27]. Among the four highly co-expressed clustered genes three genes were annotated as *FAD2* desaturases or acetylenases, and the fourth was shown to belong to five tomato homologs of *CER1 decarbonylase* [27]. In *Arabidopsis thaliana CER1* (*ECERIFERUM1*) and *CER3* (*ECERIFERUM3*) gene products catalyze fatty acid decarboxylation in the alkane biosynthetic pathway and play a major role in wax production [28]. Several orthologous genes of *CER1* and *CER3* have been identified in other plant species such as *Brassica napus*, *Camelina sativa*, and *Brachypodium distachyon* [29–31]. In carrot *CER1/CER3* family members have not been described yet.

Despite the extensive research concerning the biochemical identification, characterization and putative biological function, less knowledge exists about the genetic control of PA accumulation in carrot roots [4]. However, for breeding of carrot cultivars with a reduced bitter taste (i.e. for baby food or juice and pulp production), or with a higher pathogen resistance and enhanced health benefits more information about the inheritance of PA accumulation is needed. Genetic dissection by QTL analysis and the utilization of gene-specific (functional) molecular markers linked to QTLs for natural product biosynthesis could support such breeding strategies [32]. As a first attempt for carrot PAs, Le Clerc et al. [33] identified QTLs for the FaDOH levels in roots in some regions of a carrot linkage map and associated them with QTLs for bitterness and resistance to the fungus *Alternaria dauci*. However, no putative candidate genes were investigated in this study.

In this study, we performed a QTL analysis in a 400 plants large carrot F_2_ family derived from a cross of inbred lines of a cultivated and a wild *Daucus* accession rich in PAs. Major QTLs were identified for both root levels of FaOH and FaDOH, enabling the detection of numerous genomic regions carrying QTLs and candidate genes from the plant fatty acid metabolism. To discover putative candidate genes, we examined and extended the *FAD2* gene family in carrot and, for the first time, we used the carrot genome sequence for an inventory of *CER1/3* genes putatively involved in the genetic control of PA biosynthesis.

## Results

### Polyacetylene phenotyping and QTL mapping

**Fig. 1:**
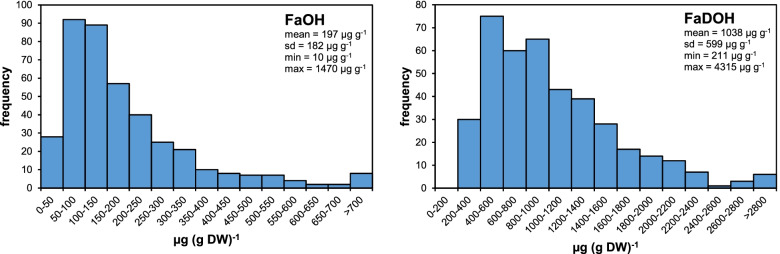
Distribution of falcarinol (FaOH) and falcarindiol (FaDOH) levels in carrot roots of 400 individuals of F_2_ progeny CA

Two main C_17_-PAs FaOH and FaDOH were quantified in carrot root samples of 400 individual F_2_ plants of the progeny ‘CA’ (P_1870 x P_345B). There was a large phenotypic variation among the F_2_ genotypes, with a population mean of 197 μg/g dry weight (DW) for the FaOH level, and an about five-fold higher FaDOH content (1038 μg/g DW) (Fig. 1). We observed few individuals with less than 30 μg/g DW FaOH and three plants with a FaOH content above 1000 μg/g. The FaDOH contents varied between 200 μg/g and 3000 μg/g in the analyzed plants (Additional file 1: Table S1). Since a major prerequisite for bi-parental QTL mapping is a cross between contrasting parental genotypes, we performed a pre-test of the parents. In average, the plants of the *D. c. commutatus-*derived parent line (P_1870) had a twice as high FaOH content compared to the parent plants of the cultivated carrot line (P_345B), and the FaDOH content was about 3.5 times higher, respectively (Additional file 2: Fig. S1).

A total of 713 SNP markers were used to construct a 505 cM linkage map for the nine carrot chromosomes based on 374 F_2_ plants of the CA progeny (Additional file 3: Fig. S2). The map constructed has an average map resolution of 1 cM with a maximum gap of 11.7 cM on chr_8. With regard to the physical position of the markers, the chromosomes are well covered with the largest gap size of 1.48 Mbp. Severe segregation distortions were noted for chromosomes chr_1, chr_5 and chr_9, all positive towards the P_345B parent. To check if the segregation distortions were caused by bolting, the bolted individuals were genotyped for the distorted regions, and only chr_1 showed a different allele distribution, showing a single incomplete recessive locus on chr_1, between 16.6–25.6 Mbp (P_1870 origin; data not shown).

**Fig. 2:**
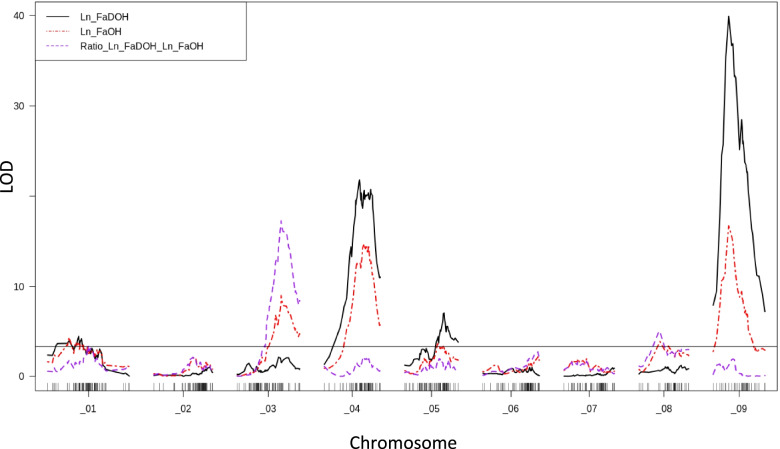
Interval Mapping’s logarithm of odds (LOD) scores of markers for PA contents and PA ratio (FaDOH/FaOH), shown alongside the nine carrot chromosomal linkage groups. The LOD threshold for significance (*p* < 0.05) calculated by 5000 permutations is shown as black line. Linkage map is presented in Additional file 3: Fig. S2

QTL analysis mapped in total 10 QTLs for the concentrations of FaOH (6 QTLs) and FaDOH (4 QTLs), and two additional QTLs were identified for the concentration ratio of FaDOH and FaOH (Table 1). The six QTLs for the FaOH concentration were located on the chromosomes chr_1, chr_3, chr_4, chr_5, chr_8 and chr_9 (Fig. 2). For FaOH the QTL FaOH_9.1 showed the highest LOD value (16.7) and explains 15.4% of the phenotypic variation. A second major QTL for FaOH with strong statistical support was found on chr_4 (LOD 14.5). For FaDOH the four QTLs were identified on chr_1, chr_4, chr_5 and chr_9 (Fig. 2). As shown by the QTL peak positions (Table 1), on chr_9 a strong QTL (LOD 40.4) explaining 24.6% of the variation was located at the same genomic position as the QTL for FaOH (*FaOH_9.1*). With regard to the QTLs on chr_4 the peak of QTL FaDOH_4.1 (LOD 21.8) showed a distance of about 4.5 Mbp to QTL *FaOH_4.1*, whereas the two QTL confidence intervals were in a similar magnitude and located in overlapping genomic regions. Analysis of QTL peak marker - trait associations showed that genotypes homozygous for the “wild” allele ‘B’ as well as the heterozygote genotypes showed significant higher PA contents (Additional file 4: Fig. S3). A major QTL (LOD 17.3) for the FaDOH/FaOH ratio (*Ratio_3.1*) was identified at the end of chr_3, and a second QTL for this parameter was located at the same position on chr_8 as the QTL *FaOH_8.1* (Table 1, Fig. 2).

**Table 1:** QTLs for FaOH and FaDOH concentrations measured in roots of carrot F_2_ progeny CA and parameters of 2-LOD confidence intervals

					Genetic 2-LOD interval (cM)			Physical 2-LOD interval (Mbp)			Markers 2-LOD interval			QTL effect (mean)^a^		
Substance	QTL	Chrom.	LOD	% Var.	Start	Peak	Stop	Start	Peak	Stop	Start	Peak	Stop	AA	AB	BB
FaOH	*FaOH_1.1*	1	4,34	5,57	4,8	22,4	53,6	824,774	540,6415	47,969,302	K0514	K4057	K1504	4,79	5,03	5,43
FaOH	*FaOH_3.1*	3	8,99	9,93	41,2	42,8	57,5	43,925,869	45,134,655	49,341,067	K1064	K1792	K0474	4,63	5,12	5,28
FaOH	*FaOH_4.1*	4	14,48	8,41	32,9	40,4	47,8	13,388,045	20.392970	33,347,895	K3873	K3654	K1382	4,70	5,21	5,76
FaOH	*FaOH_5.1*	5	3,64	6,88	17,8	33,3	53,6	524,0197	22,019928	41,938,893	K2562	K1670	mCA4358	4,75	5,11	5,09
FaOH	*FaOH_8.1*	8	3,89	6,18	8,9	21,5	51,2	200,9986	521,4614	31,631,123	K2093	K1762	K1770	4,90	4,92	5,34
FaOH	*FaOH_9.1*	9	16,71	15,35	13,0	16,1	21,8	434,2239	637,6534	11,832,494	K0850	K3482	K3568	4,50	5,17	5,59
FaDOH	*FaDOH_1.1*	1	4,45	7,28	4,8	31,9	53,6	824,774	21,266,018	47,969,302	K0514	K4090	K1504	6,72	6,78	7,29
FaDOH	*FaDOH_4.1*	4	21,81	8,51	32,9	35,5	48,4	13,388,045	15,954,204	33,764,607	K3873	K1837	K0154	6,54	6,99	7,48
FaDOH	*FaDOH_5.1*	5	7,06	8,30	35,7	39,4	46,0	25,598,051	33,366,279	39,470,106	K0871	K3010	K1404	6,61	6,83	7,15
FaDOH	*FaDOH_9.1*	9	40,35	24,55	13,0	16,1	18,9	434,2239	637,6534	849,4898	K0850	K3482	K3359	6,27	6,97	7,49
FaDOH/FaOH	*Ratio_3.1*	3	17,27	18,29	40,2	42,8	50,7	43,339,133	45,134,655	47,622,391	K0980	K1792	K1640	1,52	1,34	1,28
FaDOH/FaOH	*Ratio_8.1*	8	5,00	5,11	8,9	21,5	51,2	200,9986	521,4614	31,631,123	K2093	K1762	K1770	1,42	1,40	1,28

^a^Alleles of QTL effects: A - Parent P_345B; B - Parent P_1870

### Identification of *FAD2* and *CER1/3* genes in the carrot genome

We performed a reannotation of the whole carrot genome sequence vers.2 [34] to explore if there are carrot *FAD2* genes, which have not yet been identified in previous studies. In addition to the *FAD2* inventory of [5], who described 24 members of the carrot *FAD2* family, we detected seven undescribed putative *FAD2* gene models, which are not annotated in the carrot genome, yet (Additional file 5: Data S1). They extend the total number of *Daucus FAD2s* to 31. The total inventory of carrot *FAD2s* including the gene names used in this study is shown in Additional file 6: Table S2. The newly predicted *FAD2* genes *DcFAD2–29*, *DcFAD2–30*, and *DcFAD2–31* are located as single genes on chromosomes chr_1, chr_3, and chr_4, respectively. The other four genes (*DcFAD2–25* to *DcFAD2–28*) are clustered together with the already known *DcFAD2–4* (DCAR_027655) on chr_8 (Additional file 6: Table S2). This five-gene cluster spans a genomic region of 12.4 kb. The seven newly identified *FAD2* genes showed characteristic *FAD2* sequence motifs, notably the three highly conserved histidine-rich motifs (Histidine box) which are part of the active sites of the enzyme [20]. Based on alignment of their deduced protein sequences the consensus sequences for the three His boxes in the seven genes are H-E-C-X-H, H-X-R-H-H, and H-V-X-H-H and correspond to the His boxes of the other carrot *FAD2s* (Additional file 7: Fig. S4). Also, the conserved aromatic amino acid-rich motif Y-X-X-K/R/D/E-L/F/M/I at the C-terminus is present with one exception (*DcFAD2–29*), where the amino acid P is present at the fifth position of the motif. This motif was reported to be involved in the localization of FAD2 enzymes in the ER [35] and was recently used to support categorization of tomato *FAD2s* [25]. Therefore, based on sequence information, we suppose that the newly found genes are members of the carrot *FAD2* family. A phylogenetic analysis of the carrot *FAD2s* showed, that *DcFAD2–25*, *DcFAD2–26*, *DcFAD2–27* and *DcFAD2–28* are located in clade I (Additional file 8: Fig. S5) containing 10 known *Daucus FAD2s* which have been classified as divergent *FAD2s* [5]. All gene predictions from clade I show the amino acid G immediately preceding the first His box whereas the genes of clade II possess the amino acid A at this position and likely represent canonical FAD2 desaturases. It has been proposed that the amino acid G might indicate the functionally divergent FAD2s like acetylenases [36]. Clade I contains the genes *DcFAD2–6* (DCAR_017011), *DcFAD2–7* (DCAR_013552), and *DcFAD2–8* (DCAR_013548) which were functionally characterized as Δ12-acetylenases [5]. *DcFAD2–6* is highly similar (amino acid identity 96%) to the parsley gene *PcELI12,* which is also known as an FAD2-derived acetylenase [36]. Hence, we assume that *DcFAD2–25*, *DcFAD2–26*, *DcFAD2–27,* and *DcFAD2–28* might have an acetylenase function too. The three genes *DcFAD2–29*, *DcFAD2–30*, and *DcFAD2–31* build a separate clade III (Additional file 8: Fig. S5) but appear to be also *FAD2s* since the homology with a predicted chloroplast *ω6- fatty acid desaturase* gene (*DcFAD6_DCAR_019387)* is very low (results not shown).

**Fig. 3:**
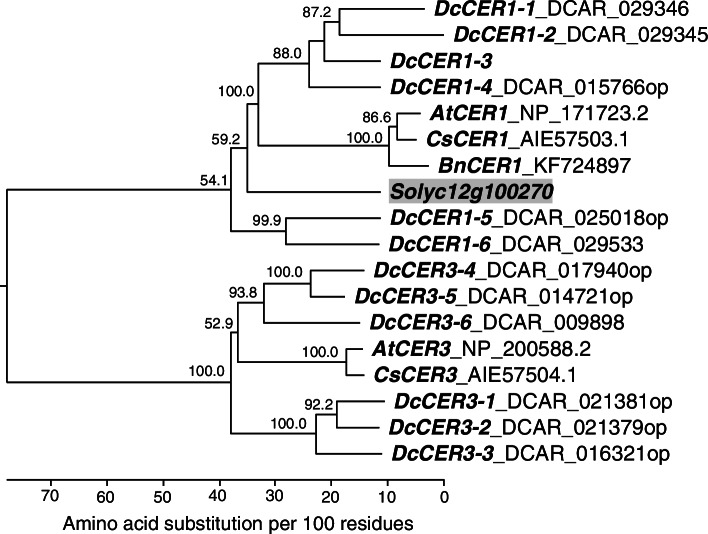
Phylogenetic tree of predicted *Daucus CER1/3* proteins (DCAR numbers according [34]; op - optimized prediction) and known plant *CER1/3 s* (*AtCER1*_NP_171723.2 and *AtCER3*_ NP_200588.2 from *A. thaliana*; *CsCER1*_KJ461885 and *CsCER3*_AIE57504.1 from *Camelina sativa; BnCER1*_KF724897 from *Brassica napus*). The putative tomato *CER1* gene *Solyc12g100270* [27] is shaded in grey. Multiple sequence alignment was performed by ClustalW using the Lasergene (DNASTAR) software package. A phylogenetic tree was constructed using the Kimura distance formula to calculate distance values and bootstrap analysis (1000 replicates). Numbers indicate bootstrap replication, and branch length is scaled below the tree indicating the number of amino acid substitutions per 100 amino acids

To compile a first inventory of carrot *CER1/3* genes, we used the predicted tomato *CER1 decarbonylase* gene *Solyc12g100270* [27] for BLAST searches in the carrot genome and in a whole plant protein database (NCBI). In total 12 putative carrot *CER1/3 s* were found in the carrot genome. Eleven gene models are already annotated in the carrot genome [34], but seven of them appeared to show uncomplete transcript sequences and were complemented by manual comparisons with other plant *CER1/3 s* and bioinformatically predicted carrot *CER1/3* gene models. The putative *CER1/3* genes are located either as single or unclustered genes on the carrot chromosomes chr_3, chr_4, and chr_5, respectively, or as two clustered genes each on chr_6, chr_7, and chr_9 (Table 2). The gene model *DcCER1–3* on chr_6 has not been annotated yet in the carrot genome sequence (Additional file 5: Data S1). The predicted *Daucus* CER1/3 proteins were compared with other plant CER1 and CER3 proteins as for instance those of *A. thaliana* [28] or *C. sativa* [29], and a phylogenetic tree was constructed which showed two major clades consisting each of six carrot *CER1* or *CER3* genes, respectively (Fig. 3). Sequence alignments of the 12 carrot gene models and other plant *CER1/3 s* showed that the three typical His boxes [37] are also present in the carrot genes (Additional file 9: Fig. S6). Two of the genes (*DcCER1–1*, *DcCER3–1*) might be truncated and functionally inactive. The highest amino acid sequence identity was 85.5% for the genes *DcCER3–4* and *DcCER3–5* and 85.3% for *DcCER3–1* and *DcCER3–2*, whereas the maximum identity among the *CER1* genes was 78.2%. The *DcCER1*s shared only 32 to 36% sequence identity with the *DcCER3* genes (Additional file 10: Fig. S7).

**Table 2:** List of *Daucus carota CER1* and *CER3* gene models sorted by their physical position on the assembled nine carrot chromosomes according to the whole genome sequence [34]

		Genomic coordinates^a^				Optimized	Predicted	CDS	No. of	Protein
Chromosome^a^	Gene Name	Strand	Start	Stop	Locus name^a^	prediction	function	length	introns	length
3	*DcCER3–6*	for	12,173,825	12,178,868	DCAR_009898	no	CER3	1899	10	633
4	*DcCER1–4*	rev	8,094,127	8,099,851	DCAR_015766	yes	CER1	1884	8	628
4	*DcCER3–5*	for	19,421,348	19,424,982	DCAR_014721	yes	CER3	1905	9	635
5	*DcCER3–3*	for	2,179,381	2,183,098	DCAR_016321	yes	CER3	1914	9	638
5	*DcCER3–4*	for	25,089,512	25,093,379	DCAR_017940	yes	CER3	1902	10	634
6	*DcCER3–1*	rev	nd	23,574,832	DCAR_021381	yes	CER3	1713	nd	571
6	*DcCER3–2*	rev	23,591,227	23,597,355	DCAR_021379	yes	CER3	1908	10	636
7	*DcCER1–3*	rev	22,041,265	22,045,133	XP_017216154.1		CER1	1863	nd	621
7	*DcCER1–5*	for	22,046,109	22,048,531	DCAR_025018	yes	CER1	1848	nd	616
9	*DcCER1–2*	rev	5,028,865	5,034,295	DCAR_029345	no	CER1	1881	9	627
9	*DcCER1–1*	rev	5,038,893	5,042,667	DCAR_029346	no	CER1	1719	7	573
9	*DcCER1–6*	rev	8,516,093	8,518,841	DCAR_029533	no	CER1	1845	6	615

^a^ Chromosomes, genomic coordinates, and locus names according the carrot whole genome sequence assembly vers.2 [34]; *nd* not determined

### Association of QTLs with candidate genes

Chromosomal start and end positions of the QTL intervals were utilized to compare the QTL regions detected with the chromosomal position of *FAD2* and *CER1/3* gene models described in the previous subsection. In total, nine out of the 12 identified QTL regions comprise *FAD2* and/or *CER1/3* candidate genes (Table 3). The number of *FAD2* candidate genes per QTL region varies between three and nine, and the number of *CER1/3* candidate genes varies from one to three, respectively. Only for the two QTLs on chr_3 (*FaOH_3.1, Ratio_3.1*) and for the QTL for FaDOH (with narrow 2-LOD interval) on chr_5 no candidate genes belonging to the analyzed gene families could be found. With regard to the major QTL for FaDOH on chr_9 (QTL peak at position 6.376534 Mbp) two putative *CER1* gene models (*DcCER1–1*, *DcCER1–2*) are located within the QTL interval which spans the region 4.342239–8.494898 Mbp. A third *CER1* gene (*DcCER1–6*) at position 8.516093 (Table 2) is located just outside the 2-LOD confidence interval. However, the support interval of the QTL for FaOH on chr_9 (4.342239–11.832494 Mbp) contains all three *CER1* genes. The physical QTL peak position chr_9 is about 1.4 Mbp distant from the two genes *DcCER1–1* (DCAR_029346) and *DcCER1–2* (DCAR_029345) and a fourth putative *CER1* gene (DCAR_029349) which is not complete. Therefore, this gene fragment (called as *DcCER1–7*) has not been considered as candidate gene and was not included in this study. *DcCER1–1* amino acid sequence showed a deletion of 36 amino acids (Additional file 9: Fig. S6), which was confirmed by amplicon sequencing (not shown). The support intervals for the QTLs for FaOH and FaDOH on chr_4 covered a comparatively large genomic region and contain the six-gene *FAD2* cluster, starting at position 29.308974 (Additional file 6: Table S2). Furthermore, close to this position there is the candidate gene *DcCER3–5* at position 19.421348, too (Table 2). The largest *FAD2* gene cluster on chromosome chr_8 consists of nine *FAD2* gene models. It is associated with minor QTLs for FaOH and QTLs for the ratio FaOH/FaDOH.

**Table 3:** Putative *FAD2* and *CER1/3* candidate genes located in 2-LOD QTL intervals. For positions of QTL intervals and candidate genes, see Table 1 (QTL intervals), Additional file 6: Table S2 (*FAD2* positions), and Table 2 (*CER1/3* positions)

QTL	Chrom.	*FAD2* candidate genes	*CER1/3* candidate genes
*FaOH_1.1*	1	*DcFAD2–3, DcFAD2–18, DcFAD2–29*	
*FaOH_4.1*	4	*DcFAD2–7, DcFAD2–8, DcFAD2–16,*	*DcCER3–5*
		*DcFAD2–17, DcFAD2–19, DcFAD2–22*	
*FaOH_5.1*	5	*DcFAD2–6, DcFAD2–11, DcFAD2–13,* *DcFAD2–20, DcFAD2–23, DcFAD2–24*	*DcCER3–4*
*FaOH_8.1*	8	*DcFAD2–1, DcFAD2–2, DcFAD2–4,*	
		*DcFAD2–9, DcFAD2–21, DcFAD2–25,*	
		*DcFAD2–26, DcFAD2–27, DcFAD2–28*	
*FaOH_9.1*	9		*DcCER1–1, DcCER1–2, DcCER1–6*
*FaDOH_1.1*	1	*DcFAD2–3, DcFAD2–18, DcFAD2–29*	
*FaDOH_4.1*	4	*DcFAD2–7, DcFAD2–8, DcFAD2–16,*	*DcCER3–5*
		*DcFAD2–17, DcFAD2–19, DcFAD2–22*	
*FaDOH_9.1*	9		*DcCER1–1, DcCER1–2*
*Ratio_8.1*	8	*DcFAD2–1, DcFAD2–2, DcFAD2–4,*	
		*DcFAD2–9, DcFAD2–21, DcFAD2–25,*	
		*DcFAD2–26, DcFAD2–27, DcFAD2–28*	

### Expression analysis of *FAD2* and *CER1/3* candidate genes

**Fig. 4:**
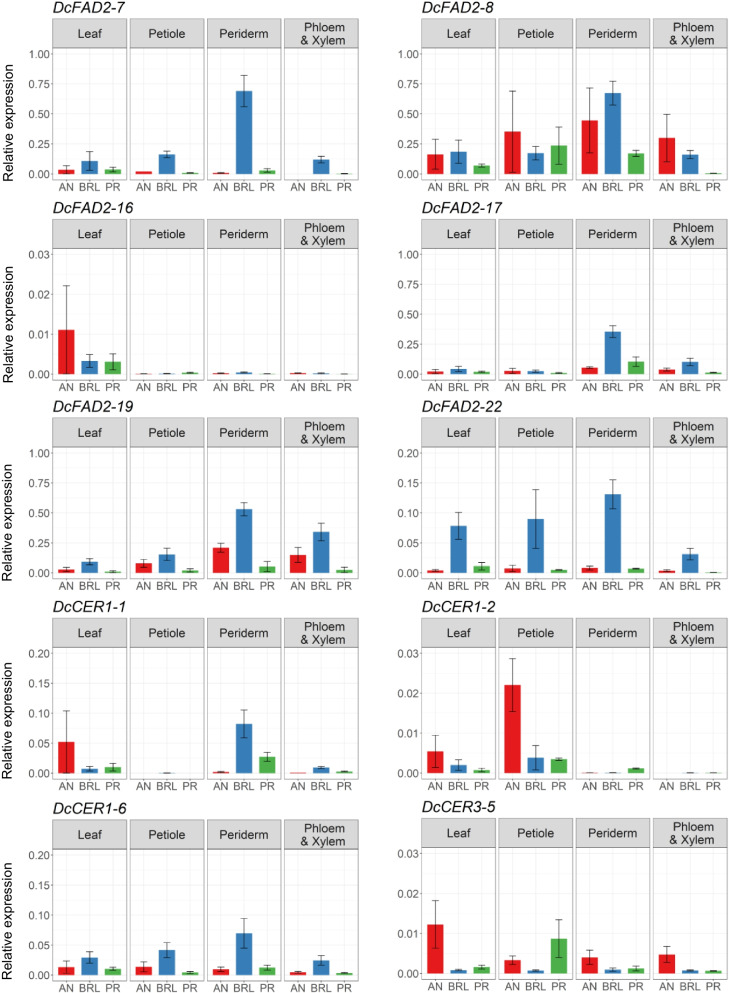
Tissue-specific expression profiles of *FAD2* and *CER1/3* candidate genes associated with major QTLs on chromosomes chr_4 and chr_9. The levels of RNA transcripts were analysed in leaf, petiole, periderm, and a mixture of phloem and xylem of Anthonina (AN), Breeding line (BRL) and Presto (PR). Data represent means of three individual plants of each cultivar with error bars indicating standard error

To study the tissue-specific expression patterns of candidate genes, expression levels of 18 genes (14 *FAD2* and *4 CER1/3* genes) were analyzed in four different tissues of three individual plants from each of the carrot cultivars Anthonina (AN), Breeding line (BRL) and Presto (PR) by qRT-PCR. Based on their location within the 2-LOD confidence intervals of strong QTLs (LOD > 10) the candidate genes *DcFAD2–7***,** *DcFAD2–8*, *DcFAD2–16*, *DcFAD2–17*, *DcFAD2–19*, *DcFAD2–22* on chromosome chr_4 and *DcCER1–1*, *DcCER1–2* and *DcCER1–6* on chr_9 were analyzed first, and their relative expression levels are shown in Fig. 4. A high expression level was observed for *DcFAD2–8* and *DcFAD2–19*, whereas transcripts were hardly detected for *DcFAD2–16*, *DcFAD2–22*, *DcCER1–1*, *DcCER1–2*, and *DcCER1–6*. For the majority of the genes, transcripts accumulate most in periderm and least in leaf tissue, with the exception of *FAD2–16* and *DcCER1–2.* They tend to be leaf-specific and petiole-specific, respectively. Among all tissues and cultivars, the highest gene expression level was found in the periderm of BRL for *DcFAD2–7*, *DcFAD2–8*, *DcFAD2–17*, *DcFAD2–19*, *DcFAD2–22*, *DcCER1–1* and *DcCER1–6*. BRL showed higher expression levels for *DcFAD2–7*, *DcFAD2–19*, *DcFAD2–22* in all tissues than in the corresponding tissues of AN and PR. For *DcFAD2–8* and *DcFAD2–19* in the periderm and the mixture of phloem and xylem, expression levels were highest in BRL and lowest in PR, which correspond with the levels of average PA contents in BRL (high) and PR (low), respectively (Additional file 11: Fig. S8).

For *DcFAD2–4* associated with the minor QTL on chr_8, the transcript levels showed the same pattern as for *DcFAD2–8* and *DcFAD2–19* located within the support interval of the strong QTL on chr_4, and the expression in the periderm was highest among all three cultivars (Additional file 12: Fig. S9). For the genes which are not associated with QTLs, such as *DcFAD2–5* and *DcFAD2–12*, expression levels are high in periderm and low in leaf and petiole. However, *DcFAD2–12* was the only analysed *FAD2* gene which had a higher expression level in the vascular system (mix of phloem and xylem) compared to the expression level in the periderm.

To get an impression about the genotypic variability of candidate gene expression patterns in different individuals of the parental lines, we analyzed the relative expression of the *FAD2* and *CER1* candidate genes located on chr_4 and chr_9 in each six individuals of parental lines P_1870 and P_345B. The total RNA was isolated from the ‘PPX’ samples (mixed periderm, phloem and xylem tissues) from the individual root harvested for each genotype. For P_1870 individuals, the xylem was excluded due to lignification. The relative transcript levels were highly differentiated among the individuals even within the parental lines indicating a large genetic diversity for this trait in the parental lines (Additional file 13: Fig. S10). Similar to the tissue-specific expression study, transcripts of *DcFAD2–16*, *DcFAD2–22*, *DcCER1–1*, *DcCER1–2* were barely detected in all tissues. The individuals which have a high expression level of *DcFAD2–8,* expressed *DcFAD2–7, DcFAD2–17 and DcCER1–6* in a high level as well.

## Discussion

### Polyacetylene quantification in a carrot F_2_ family

In the present study a carrot F_2_ family derived from a cross of a cultivated carrot breeding line and the wild relative *D. c. commutatus* was used for quantification of major PAs FaOH and FaDOH. Since the roots of the original parental plants were not available for PA analysis, we measured the PA contents of each six siblings raised for the pilot experiment with the aim to identify parental lines differing in PA contents and to justify a bi-parental QTL approach. The average PA contents in the wild parent appeared to be considerably higher than the average PA contents in the cultivated breeding line. In the rare PA studies based on carrot wild relatives it was shown that *Daucus* species and subspecies can contain much higher PA contents than cultivated carrot forms [9]. Accessions of *D. c*. *commutatus* were among the (sub)species with the highest FaOH and FaDOH contents [10]. PA data collected from the pilot experiment as well as those from the main experiment showed that - with the exception of three plants - all investigated plants possessed a higher FaDOH level compared with FaOH. According to Czepa and Hofmann [7] the most abundant PA in cultivated orange carrots is FaDOH, and the highest total PA levels were found in the periderm tissue [5, 9, 38]. In our study we focused for QTL analysis on total FaOH and FaDOH contents measured in samples representing a mixture of the different root tissues (periderm, phloem, xylem). Considering the large number of F_2_ individuals involved in this study, this approach appeared to be appropriate to detect QTLs relevant for carrot breeding. A further dissection of the root tissues for QTL analysis might be applied in future studies to reveal tissue-specific changes in PA biosynthesis.

A large phenotypic variability was observed for the contents of both PAs in the F_2_ population indicating that the selection of the crossing parents was appropriate for the bi-parental QTL approach. Carrot is a highly heterozygous species, and genetic uniformity even in advanced breeding lines appears to be not the rule, as it is demonstrated by the phenotypic variation of the PA contents in the six individuals of the 345B parental line. The observed frequency distributions in the F_2_ progeny indicate a polygenic inheritance of the PA production in carrot. This is not surprising considering the complex biochemical pathway from which the falcarinol-type PAs derive. Nevertheless, the high phenotypic variation in the F_2_ family, which contains plants with no measurable contents of PAs and on the other side plants with extremely high contents above 1000 μg/g FaOH or even 3000 μg/g FaDOH suggest, that major structural or regulatory genes are involved in the control of PA biosynthesis. Generally, the inheritance of PA concentrations and distribution patterns are poorly understood. The only study on carrot PAs based on a segregating population showed that PA contents are a heritable trait [33]. In the carrot F_2_ family used in the 2-years experiment for QTL analyses of falcarinol-type PAs, a comparatively low impact of the environment on the accumulation of PAs was found, and the broad-sense heritability for, e.g. FaDOH was estimated with 0.88. Transgressive segregation was also observed in this study [33].

### Linkage mapping and QTL analysis

Carrot belongs to the vegetable species for which already several partial or saturated linkage maps have been developed and published (for review, see [39]). For the current study publicly available SNP markers [40] were used for linkage mapping, allowing integration with other published genetic maps. Overall the genetic map is smaller compared to the first high-resolution SNP-based carrot map published by Cavagnaro et al. [41], but the marker order is similar, giving no indication of regions with recombination suppression. It has to be noted that several regions of segregation distortions were identified, which could have an effect, though limited, on the general lower number of observed recombinations in this study. While segregation distortion does not affect QTL mapping especially in dense marker maps, it may lead to erroneous interpretations of the number of loci controlling a trait of interest [42].

In the present study we dissected the genetic basis of two key PAs, FaOH and FaDOH, by QTL analysis. Several major genomic regions seem to be involved in PA production in carrots. Overall, QTLs were localized on six out of the nine carrot chromosomes, which is a sign for the complexity of the genetic control of PA accumulation in carrot. Strong QTLs for FaOH and FaDOH (LOD > 10) were identified with overlapping 2-LOD confidence intervals on chromosomes chr_4 and chr_9 indicating a major involvement of these regions in PA biosynthesis. We propose that these QTL regions include major genes that control the primary or secondary PA metabolism in carrot. The QTL with the strongest statistical support was found for FaDOH in the upper region of chr_9. Interestingly, in the study of Le Clerc et al. [43] the biggest QTL for FaDOH was also located on chr_9, but according to their linkage map of chr_9 the QTL was on the opposite side. Due to the usage of proprietary markers used by these researchers (V. le Clerc, pers. comm.) it was not possible to verify the orientation of chr_9. However, it seems possible that both strong QTLs for FaDOH share a common biological origin. The QTL identified by us for both PAs in the lower region of chr_4 could be the same QTL identified for FaDOH by Le Clerc et al. [43] in this genomic region. A QTL for falcarindiol-3-acetate, a minor PA probably metabolized from FaDOH, was detected by latter researchers on top of chr_8, where we identified a QTL for the FaDOH/FaOH ratio. QTLs for PA ratio might be useful to identify loci controlling the interconversions among the PAs. It is presumed, that FaDOH is produced from the precursor substance FaOH [4, 6]. As the QTL effects of allele ‘B’ from the wild parent P_1870 are responsible for a lower PA ratio in ‘BB’ genotypes, it is probably that the QTLs for the PA ratio indicate impaired conversion of FaOH into FaDOH (i.e. accumulation of FaOH). The generally increasing QTL effects of the ‘B’ allele for all other QTLs associated with total amounts of single PAs indicate that rather the primary metabolism of common fatty acid precursors, as for instance crepenynic and dehydrocrepenynic acid, is controlled by genetic factors underlying these QTLs. Likely there was a selection during the domestication process against unfavourable “wild” alleles associated with high FaDOH contents to avoid a too high level of bitterness in cultivated carrots.

Overall, the results from our QTL analysis confirmed the utility of a carrot family derived from a wide cross of a cultivated carrot with a *D. c. commutatus* accession rich in PAs. Previous studies based on the genetics of carotenoid accumulation used a mapping population derived from a cross between a cultivated orange carrot and a wild white carrot collected in North America [44, 45]. Important advances in the understanding of the genetic control of carrot anthocyanin pigmentation have been made by using a purple wild carrot parent from Turkey [41, 42].

To dissect the genetic basis of a quantitative trait, such as the content of a natural product, two main methods have been used in the past, bi-parental QTL mapping and genome-wide association studies (GWAS). Conventional QTL mapping depends on a diverging genetic diversity of two parents and is very time-consuming since in several crop plants including carrot a F_2_ mapping population has to be developed. Moreover, QTL regions can be quite large and may include many potential candidate genes. Nevertheless, this method has been used in carrot research and breeding to elucidate the genetics of important secondary metabolites determining root quality such as β-carotene, anthocyanins, polyacetylenes, and volatile terpenes [32, 33, 41, 44]. GWAS can overcome the limitations of bi-parental QTL mapping and has great potential for detection of QTLs with high resolution in diverse sequenced genotypes. Combinatorial approaches are also very useful to compensate the limitations of each method. Both QTL analysis and GWAS have been used for the detection of *DcTPS54*, a sabinene synthase gene putatively involved in carrot flavour [32, 46] and discovered the *Or* gene controlling carotene accumulation [47, 48]. GWAS aimed at PA compounds in large sets of carrot cultivars, land races and wild relatives would allow the identification of additional QTLs, but might be especially helpful to narrow down the QTL intervals for both FaOH and FaDOH on chromosome chr_4 allowing the more precise association with candidate genes.

### Candidate genes associated with QTLs

In this work, the combination of a PA metabolite quantification, bi-parental QTL analysis and the discovery of putative candidate genes from two different gene families involved in fatty acid metabolism have been used to get insights into the genetic control of PA biosynthesis. Few biochemical pathways have been studied in carrot by a combinatorial approach based on linkage mapping, QTL analysis and candidate gene identification. In contrast to previous investigations on carrot carotenoids, anthocyanins and terpenes (for review, see [49–51]), no studies have been, to date, reported on comparable candidate gene approaches for PA compounds. The reason is, that in higher plants little is known on the genetic control and the enzymes involved in the biosynthesis of these compounds. Until recently, no carrot genes involved in PA production have been described. Work in the past on parsley (*Petroselinum crispum,* Apiaceae) identified a divergent form of FAD2 that was upregulated in response to pathogen attack and, when expressed in soybean embryos, resulted in the production of crepenynic acid and dehydrocrepenynic acid [36, 52]. These results of the parsley studies are consistent with a pathogen-responsive, divergent FAD2-mediated pathway leading to the accumulation of acetylenic fatty acids needed for PA formation [5]. The *FAD2* inventory work of Busta et al. [5] resulted in the identification of 24 carrot *FAD2* members, and it was shown after functional analysis of six genes in yeast and *Arabidopsis thaliana* that these genes represented the major entry point into carrot PA biosynthesis. We performed a reannotation of the carrot genome sequence [34] and detected seven new *FAD2* gene models, which increases the total number of carrot *FAD2* family member to the large total number of 31. It is a striking feature that carrots seem to contain the highest number of *FAD2* family members reported so far in the plant kingdom. Knowledge about the *FAD2* family including the divergent forms is based largely on research on *A. thaliana* and on oil seed crops. Despite the evidence for functional diversification of FAD2 in multiple plant families, the potential of such diversification in major crops including possible roles in biotic stress resistance has been largely unexplored [25].

Recently a biosynthetic gene cluster was discovered in tomato that was required for FaDOH production. Interestingly, among the four highly co-expressed clustered genes there was a *CER1 decarbonylase* [27]. To our knowledge, this was the first time that a possible *CER1* gene function was reported that is different from the so far recognized function in the alkane biosynthetic pathway. The *Arabidopsis* CER1 protein is known as a decarbonylase that converts fatty acid metabolites into alkanes. Alkanes are components of waxes in the plant cuticle, a waterproof barrier serving to protect land plants from both biotic and abiotic stimuli [53]. We searched for carrot orthologous genes and found six putative *CER1* genes showing a close phylogenetic relationship with the previously characterized tomato *CER1* and additionally six putative *CER3* gene models. Few reports are available about *CER* gene families in plants. Characterization of the *CER1* family genes in *Brachypodium distachyon* identified eight *CER1* homologs [31], and in rice seven putative *CER1* paralogs were identified [54].

We identified two genomic regions on two different carrot chromosomes around QTLs for FaOH and FaDOH with high LOD values of > 14 and > 21, respectively, that contain several *FAD2* and *CER1/3* genes. The six *FAD2* genes clustering on chr_4 and the three *CER1* genes on chr_9 are preliminary considered as candidate genes, based on their location within 2-LOD QTL confidence intervals. However, a causal relationship between *CER1* genes and the production of FaOH and FaDOH remains to be biochemically explained. Concerning the six *FAD2* genes associated with the major QTLs on chr_4 their distance to the QTL main peak is larger. However, in the upper region of this chromosome (outside of the 2-LOD interval) the gene *DcFAD2–31* is located which might have an influence on the calculated QTL peak positions. The six carrot *FAD2* genes associated with the strong QTL on chr_4 reside close to one another within a small region (29.3–29.4 Mbp) and might have been originated from local tandem duplications, as data from microsyntenic and phylogenomic analyses suggest [5]. This cluster also contains the two genes *DcFAD2–7* and *DcFAD2–8* which have been functionally characterized as Δ12-fatty acid acetylenases, and additionally the gene *DcFAD2–19* which was described as a bifunctional enzyme capable of catalysing both Δ12 desaturation of oleate and Δ14 desaturation of crepenynate [5]. Interestingly, in the plants of BRL which showed high PA levels in comparison to other carrot cultivars, a co-expression seem to have occurred in the periderm tissue samples for five out of the six *FAD2* candidates including *DcFAD2–7, DcFAD2–8,* and *DcFAD2–19*. In the study of [5] the highest coefficients for co-expression with *DcFAD2–19* were found for the two putative acetylenase genes *DcFAD2–7* and *DcFAD2–8.* Busta et al. [5] analysed tissue specific gene expression by qPCR in the orange cultivar ‘Danvers’ and showed that expression of *DcFAD2–7* and *DcFAD2–8* was highest in the periderm, which is in accordance with our results. Also the putative Δ12 desaturase gene *DcFAD2–11* and the functionally characterized Δ12 acetylenase gene *DcFAD2–6* which are associated with a QTL for FaOH on chr_5, showed highest transcript levels of peridermal tissues in both studies. Taken together, it is likely that the QTL region on chr_4 plays a major role in carrot PA production. Nevertheless, the situation that seven out of the 12 QTLs are associated with *FAD2* candidate genes, support the suggested importance of the *FAD2* family on the PA biosynthesis in Apiaceae. Further functional and biochemical studies are needed to identify the most relevant *FAD2s*. Even beyond identifying *FAD2* and *CER* candidate genes for QTLs identified in this study, more genes involved in PA biosynthesis might be identified elsewhere in the carrot genome. Since the PAs in this study are related in terms that they represent compounds produced at different steps in the same biosynthetic pathway, it is also possible that a single regulatory gene like a transcription factor affects the compounds produced in this pathway.

### Implications for carrot breeding

Bitterness is considered as an undesirable taste of carrot roots, which can cause consumer rejection and is one of the main reasons for low preference scores in sensory evaluations of carrots [13]. It is a very complex quality trait because numerous chemically different compounds may contribute to bitter taste. Potential bitter compounds in carrot are volatile mono- and sesquiterpenes, PAs, phenylpropanoids, and isocoumarins. Schmid et al. [8] recently listed 14 known bitter off-taste compounds including the major PAs FaOH and FaDOH. In the study of Le Clerc et al. [33] the total PA content was closely related to bitterness, and the highest quantities accumulated in the most bitter genotypes, whereas the lowest amounts were measured in the least bitter genotypes. There is some evidence from quantitative chemical analyses combined with sensory analysis that FaDOH is highly correlated with bitterness, whereas FaOH is not [7, 13]. On the other hand, several in vitro studies have confirmed that FaOH is one of most cytotoxic PAs in Apiaceae vegetables and, according the current state of knowledge, has a higher bioactivity than FaDOH [19]. A better understanding of the genetics of the PA levels present in carrot roots might support breeding carrot cultivars with low bitterness but high health potential for the consumers. To reach this goal it will be necessary to reveal the genes that control the decisive steps in the complex PA biosynthesis pathway, i.e. the formation of FaDOH. Carrot chemotypes with acceptable amounts of bioactive PAs may contribute significantly to the known positive effects of carrots on human health.

## Conclusions

The aim of the current study was to dissect the complex genetic control of PA accumulation in carrot roots. We used a carrot F_2_ family derived from a carrot wild relative rich in PAs for SNP-based QTL mapping and detected several major QTLs for FaOH and FaDOH with high LOD values on chromosomes 4 and 9. To study the association of these QTLs with *FAD2* candidate genes known to be involved in PA biosynthesis, we compared the genomic positions of *FAD2* gene models with QTL intervals and found significant relationships, such as the putative involvement of a known and partly functionally characterized six-*FAD2* gene cluster located on carrot chromosome 4 which contain acetylenase genes. To discover other putative candidate genes from the plant fatty acid metabolism, we performed a first inventory of *CER1* genes which were recently reported to be putatively involved in the production of FaDOH in tomato. The genetic association of three putative *CER1 decarbonylase* candidates with the strongest QTLs on chromosome chr_9 might be a first indication for this hypothesis. Our finding, that more or less functionally characterized carrot genes from the plant fatty acid metabolism are significantly associated with major QTLs for key PAs, will facilitate future functional gene studies and a further dissection of the genetic factors controlling PA accumulation. The results of this research are considered to have a positive impact on carrot breeding programs aiming at lowering or increasing PA concentrations and PA patterns, dependent on the requirements of the consumers.

## Methods

### Plant material and sample preparation

F_2_ population P11054 (called in this study ‘CA’) was derived from a single F_1_ plant out of a cross between a cultivated carrot inbred line ‘345B’ (parent ‘P_345B’; proprietary BASF) and an inbred from *Daucus carota* ssp*. commutatus* (parent ‘P_1870’). Both parental inbred lines were produced at Nunhems Netherlands BV (Nunhem, The Netherlands). Seeds from the original *D. c. commutatus* accession no. JKI-1870 are available from the carrot seed repository of Julius Kühn-Institute (JKI) upon request (corresponding author). The F_2_ population CA was chosen based on a pilot test with PA content measurements (spring 2020), including the parental lines (6 individuals each) and a small subset of 9 individuals from F_2_ population CA.

For the main experiment F_2_ population CA was sown in the field in May 2020, grown during the summer and harvested in October 2020 at the BASF research station in Nunhem (Limburg, Netherlands). About 15% of the plants in the field showed to bolt. A total of 550 F_2_ individuals were evaluated in the field and 400 non-bolted F_2_ individuals were selected for further PA content measurements. Next 80 bolted F_2_ individuals were sampled, but excluded from further analysis, as the roots were less developed, possible hampering reliable PA content measurements. For PA analysis of the F_2_ plants three plugs were excised from an individual carrot root at top, middle and bottom position using a cork borer (∅ 8 mm, depth 3 mm). These three plugs were then pooled for PA analysis. Plant material was shock frozen in liquid nitrogen immediately after harvest and stored at − 80 °C until freeze-drying.

A pilot test with PA content measurements was carried out to identify parental lines differing in PA content and to prove if there is sufficient phenotypic variability amongst the individuals of the F_2_ progeny. For the pilot test a slightly modified sampling method called ‘PPX’ was used, because some roots of the parental line P_1870 were extremely thin (Additional file 2: Fig. S1) and therefore it was not possible to sample a 3 mm-thick and 8 mm-in-diameter plug for these roots. For PPX sampling the root was first cut longitudinally into two halves and the PPX sample (a plug cylinder consisted of periderm, phloem and xylem from a half of the root) was taken by a 8-mm cork-borer. The samples were shock frozen in liquid nitrogen immediately after harvest and stored at − 80 °C until PA analysis and expression analysis of candidate genes.

### Analysis of polyacetylenes by HPLC/DAD

Deep-frozen root plugs (main experiment) or PPX samples (pre-test) were freeze-dried for 4 days (Christ Gamma 1–16 LSC, condenser temperature − 50 °C, pressure 0.04 mbar). Freeze-dried plugs of individual roots were precisely weighed into 1.5-mL polypropylene centrifuge tubes (approx. 20–30 mg dry weight). After addition of three steel balls (∅ 3 mm) plugs were homogenized using a mixer mill (Retsch MM 400, 30 Hz, 2 × 60 s). After homogenization 50 μL internal standard solution (0.2 g L^− 1^ *N*-vanillylnonamide in MeOH) and 300 μL acetone were added. The mixture was homogenized once again using a mixer mill (30 Hz, 60 s, room temperature), sonicated (ElmaSonic P, 37 kHz, 100 W, 5 min, 20 °C) and shaken (2400 min^− 1^, 10 min, room temperature). After centrifugation (13,000 *g*, 5 min, 20 °C) a 200-μL aliquot of the supernatant was transferred into an HPLC vial with micro-insert and stored at 6 °C until analysis.

Polyacetylene analyses were performed on an 1100 Series HPLC system (Agilent Technologies) comprising a degasser (G1322A), a binary pump (G1312A), an autosampler (G1329A), an autosampler thermostat (G1330A), a column compartment (G1316A) and a diode array detector (G1315A). Extracts (injection volume 2.5 μL) were separated on a Zorbax Eclipse XDB-C18 column (3 mm × 150 mm, 3.5 μm particle size, Agilent Technologies) using water and acetonitrile as eluent A and B, respectively. The following binary gradient program at a flow rate of 1 mL min^− 1^ was used: 0–10 min, linear from 50 to 80% B; 10–10.5 min, linear from 60 to 100% B; 10.5–13 min, isocratic, 100% B; 13–15 min, isocratic 50% B. The column and autosampler temperature was maintained at 40 °C and 6 °C, respectively. The diode array detector response time was set at 0.2 s, the optical slit width at 4 nm. Polyacetylenes were detected at 196 nm with a spectral bandwidth of 4 nm, the internal standard *N*-vanillylnonamide at 204 nm with a spectral bandwidth of 4 nm. ChemStation software (version B.03.02) was applied for controlling the instrument, data acquisition and quantitative analysis. *N*-Vanillylnonanamide (t_R_ 2.53 min), falcarindiol (t_R_ 5.09 min) and falcarinol (t_R_ 9.48 min) were quantified based on peak area using external standard calibration method. Therefore, the following calibration curves were established: (i) *N*-vanillylnonamide: calibration range 1–600 ng, 14 points, linear regression model (y = m x), equal weighting, R^2^ = 0.99987; (ii) Falcarindiol: calibration range 5–500 ng, 9 points, linear regression model (y = m x), equal weighting, R^2^ = 0.99996; (iii) Falcarindiol: calibration range 500–1000 ng, 6 points, logarithmic regression model (y = m ln(x) + b), equal weighting, R^2^ = 0.99919; (iv) Falcarinol: calibration range 5–1000 ng, 14 points, linear regression model (y = m x), equal weighting, R^2^ = 0.99970. Polyacetylene levels were corrected using the recovery rate of the internal standard.

### Construction of a SNP-based linkage map and QTL analysis

DNA of 400 CA F_2_ plants was extracted from dried young leaflets using LGC/SBeadex^tm^ plant DNA purification kit (standard protocol). Genotyping was performed with a proprietary SNP array. A dataset, comprised of 374 F2 individuals and 705 polymorphic literature markers [40] and 8 proprietary SNP markers, was used for genetic mapping. The SNP scores were translated to parental scores, with the A genotype for homozygous P_345B parent allele, B genotype for homozygous P_1870 parent allele, H for heterozygous genotype and U for undetermined genotype. For construction of the genetic linkage map Joinmap 5.0 software [55] was used with standard settings. Markers were grouped at a recombination frequency threshold of 0.25 into nine linkage groups, which were renamed to their representing chromosomes (chr_1 until chr_9). Maximum Likelihood (ML) mapping was used to calculate the genetic distances. After the calculation overlapping genetic positions were pruned, leaving a genetic map only represented with unique genetic positions. The QTL analysis was conducted in R 3.6.2, using R/qtl package [56]. Interval Mapping (IM) and Composite Interval Mapping (CIM) were used for a preliminary QTL detection, followed by a “scantwo” two-dimensional scan to detect possible QTL interactions. For each analysis, 5000 permutations were performed (n.perm = 5000). The significance level was set to 95% (alpha = 0.05). Final QTL models were made with FitQTL and LODINT with a 2-LOD drop was used to determine the confidence intervals.

### Discovery of *FAD2* and *CER1/3* candidate genes

The homology-based gene prediction program GeMoMa (version 1.6.beta, [57]) was used to reannotate the published carrot genome [34] and to determine potential *FAD2* candidate genes in carrot, which have not yet been previously described. Totally 12 genomes (*Arabidopsis thaliana* (TAIR10), *Brachypodium distachyon* (v3.1), *Glycine max* (Wm82.a2.v1), *Lactuca sativa* (v5), *Mimulus guttatus* (v2.0), *Oryza savita* (v7.0), *Prunus persica* (v2.1), *Populus trichocarpa* (v3.1), *Sorghum bicolor* (v3.1.1), *Setaria italica* (v2.2), *Solanum lycopersicum* (v2.5), *Theobroma cacao* (v1.1)) were used as reference with the software parameters described in [32]. The newly annotated carrot genome was used for BLASTP searches based on the putative 24 members of the carrot *FAD2* family [5]. For a first approach to identify putative carrot *CER1/3* genes the annotated tomato gene *Solyc12g100270* predicted as putative *CER1 decarbonylase* [27] was used for BLAST of the carrot genome vers.2 [34] by Phytozome vers.13 [58]. All identified putative *CER1/3* sequences annotated in the carrot genome were used for a BLASTP search at NCBI using the non-redundant protein sequence database. Predicted carrot *CER1/3* protein sequences and known plant *CER1/3* sequences were used for comparisons with the original DCAR sequences. In case that the DCAR locus appeared to be incompletely annotated, the DCAR gene model was edited by adding the missing regions (optimized prediction).

### Expression analysis of *FAD2* and *CER1/3* candidate genes

The tissue-specific expression analysis of candidate genes was carried out with the purple cultivar ‘Anthonina’ (AN; Seminis, USA), the orange cultivar ‘Presto’ (PR; Vilmorin, France) and a selected orange carrot breeding line (BRL; Nunhems BV, Netherlands). For the expression study seeds were sown and cultivated in 19 cm/30 cm W/H plastic pots in a sand-humus mixture (v/v 3/1) under optimized greenhouse conditions at 25/20 °C D/N and 16 h photoperiod until harvest. Samples from leaves, petioles, root periderm and mixed root phloem/xylem were taken from three individual plants about 100 days after sowing, immediately shock frozen and stored at − 80 °C until RNA isolation. Total RNA was isolated from from leaf, petiole, periderm, and the mixture of phloem and xylem of carrot cultivars AN, BRL, and PR. RNA was extracted from 80 to 100 mg plant material utilizing innuPREP PlantRNA Kit (Analytik Jena, Jena, Germany) with an additional DNAse treatment by innuPREP DNase I Digest Kit (Analytik Jena), according to the manufacturer’s instructions. First-strand cDNA was synthesized by Revert-Aid First Strand cDNA Synthesis Kit (Thermo Scientific, Massachusetts, USA) following the manufacturer’s instructions. The Real-Time Quantitative Reverse Transcription PCR (qRT-PCR) reactions were performed in C1000 Touch™ Thermal Cycler (Bio-Rad Laboratories, California, USA). The amplification was carried out in duplicate in 10 μl reaction volumes consisting of 1× iTaq SYBR Green Supermix (Bio-Rad), 0.4 μM each primer and 1 μl cDNA. Primers were designed using NCBI Primer-BLAST tool (https://www.ncbi.nlm.nih.gov/tools/primer-blast/). The information for the primer used for this study is listed in Additional file 14: Table S3. The qPCR thermal cycling conditions were as follows: initial denaturation at 95 °C for 5 min, followed by denaturation for 5 s, annealing and extension at 60 °C for 30 s for 40 cycles. The data from three individual plants were normalized to the housekeeping genes *actin1* (*ACTIN*), *elongation factor* (*EF-1α*) and *poly-ubiquitin* 10 (*UBQ*) [59]. The relative gene expression levels were calculated using the delta Ct method with calculated primer efficiencies [60].

## Supplementary Information

**Additional file 1: Table S1.** PA contents of 400 F_2_ individuals of the population CA.

**Additional file 2: Figure S1.** Root phenotypes and PA contents of parents.

**Additional file 3: Figure S2.** Linkage map of population CA.

**Additional file 4: Figure S3.** Relationship of QTL peak marker genotypes with PA contents.

**Additional file 5: Data S1.** Coding sequences (CDS) and predicted proteins of new *FAD2s* and *CER1s.*

**Additional file 6: Table S2.** List of *FAD2* gene models and their genomic positions.

**Additional file 7: Figure S4.** Alignment of predicted carrot *FAD2* protein sequences.

**Additional file 8: Figure S5.** Phylogenetic tree of carrot *FAD2* protein sequences.

**Additional file 9: Figure S6.** Alignment of predicted carrot *CER1* and *CER3* protein sequences.

**Additional file 10: Figure S7.** Amino acid identity of carrot *CER1* and *CER3* proteins.

**Additional file 11: Figure S8.** PA contents of cultivars AN, BRL and AN.

**Additional file 12: Figure S9.** Tissue-specific qRT-PCR of additional *FAD2* genes in 3 cultivars.

**Additional file 13: Figure S10.** qRT-PCR of candidate genes associated with major QTL in parents.

**Additional file 14: Table S3.** qRT-PCR primer information.

## Data Availability

Seeds of the original *D. c. commutatus* accession no. JKI-1870 are available from the carrot seed repository of Julius Kühn-Institute (JKI) upon request (contact: corresponding author). All data generated or analysed during this study are included in this published article and its supplementary information files. The predicted sequences of eight gene models *DcFAD2–25* to *DcFAD2–31* and *DcCER1–3,* which are not yet annotated in the current carrot genome vers.2 [34], are included in the supplement of this article (Additional file 5: Data S1).
