# Supplementary material for: The genetic control of polyacetylenes involved in bitterness of carrots (Daucus carota L.): Identification of QTLs and candidate genes from the plant fatty acid metabolism
Source: BMC Plant Biol. 2022 Mar 2;22:92. doi: 10.1186/s12870-022-03484-1 (PMC8889737; doi:10.1186/s12870-022-03484-1)
Supplement: Supplementary file 2 — Additional file 2: Figure S1. Root phenotypes and PA contents of parents. [file 12870_2022_3484_MOESM2_ESM.pdf]

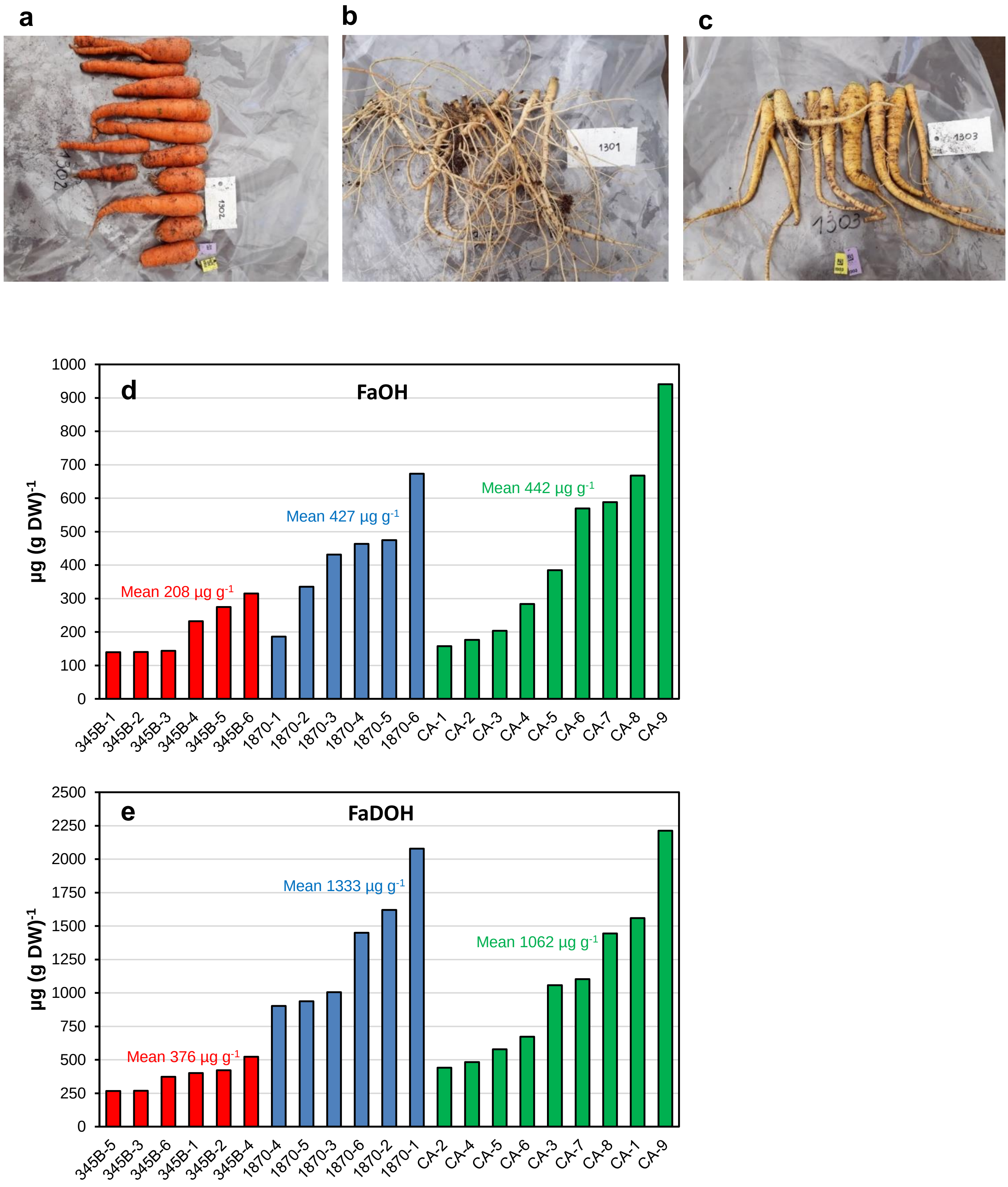

Figure S1 Characteristic carrot root phenotypes of the cultivated inbred line ´345B´ (**a**), of the inbred line derived from *D. carota* ssp. *commutatus* accession JKI 1870 ('1870') (**b**) and of the F_2 population ´CA´. FaOH and FaDOH levels of each six parental individuals of ´345B´ and´1870´, and nine F_2 individuals of ´CA´ are shown in **d** and **e**, respectively. Due to morphological reasons, root tissue for PA analysis was sampled using the 'PPX' method. For details, see method section.
