# Supplementary material for: The genetic control of polyacetylenes involved in bitterness of carrots (Daucus carota L.): Identification of QTLs and candidate genes from the plant fatty acid metabolism
Source: BMC Plant Biol. 2022 Mar 2;22:92. doi: 10.1186/s12870-022-03484-1 (PMC8889737; doi:10.1186/s12870-022-03484-1)
Supplement: Supplementary file 3 — Additional file 3: Figure S2. Linkage map of population CA. [file 12870_2022_3484_MOESM3_ESM.pdf]

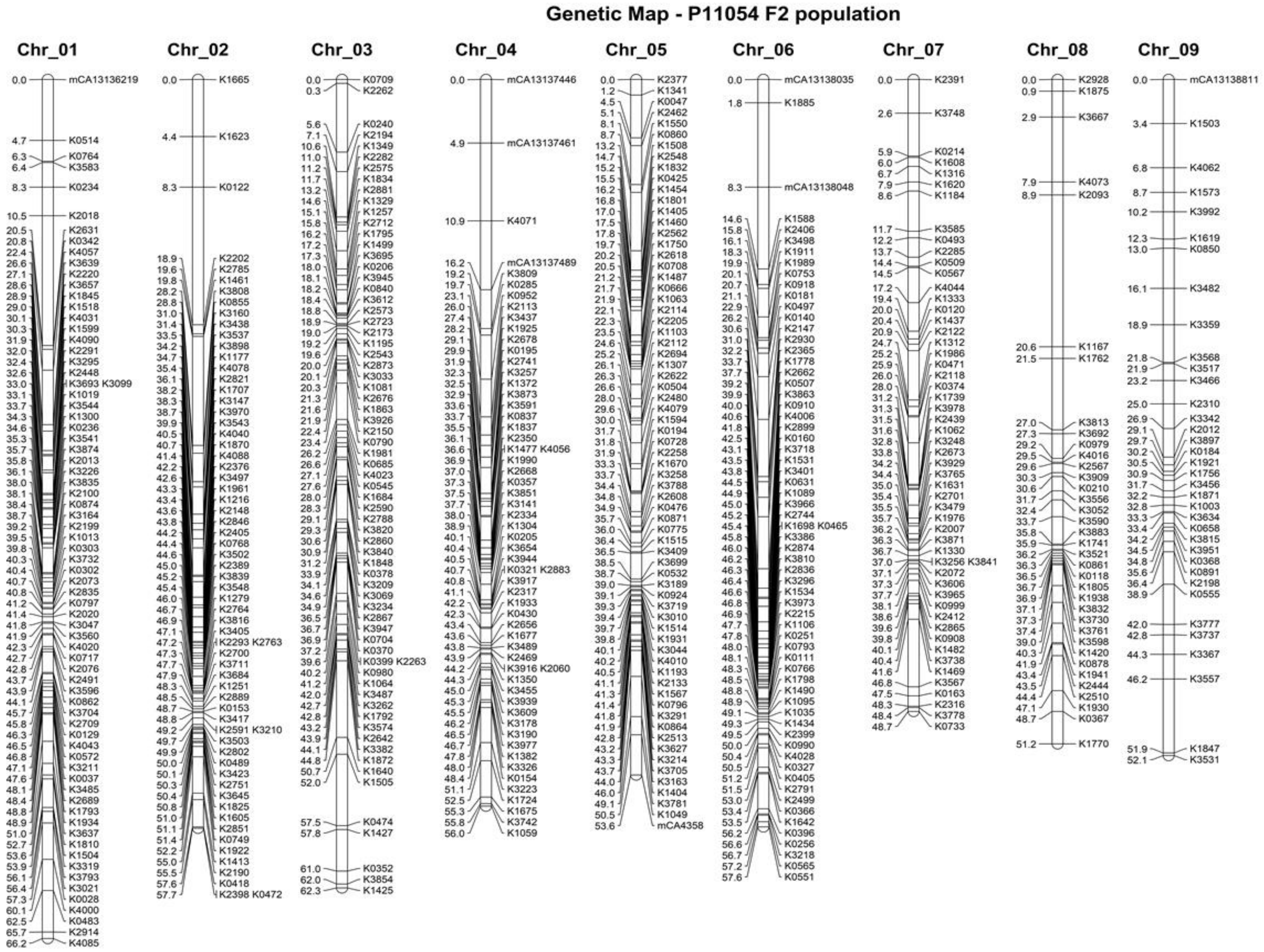

Figure S2 Genetic map of F_2 population P11054 (called CA in this study), based on 374 individuals and 526 SNP markers
