# Supplementary material for: The genetic control of polyacetylenes involved in bitterness of carrots (Daucus carota L.): Identification of QTLs and candidate genes from the plant fatty acid metabolism
Source: BMC Plant Biol. 2022 Mar 2;22:92. doi: 10.1186/s12870-022-03484-1 (PMC8889737; doi:10.1186/s12870-022-03484-1)
Supplement: Supplementary file 4 — Additional file 4: Figure S3. Relationship of QTL peak marker genotypes with PA contents. [file 12870_2022_3484_MOESM4_ESM.pdf]

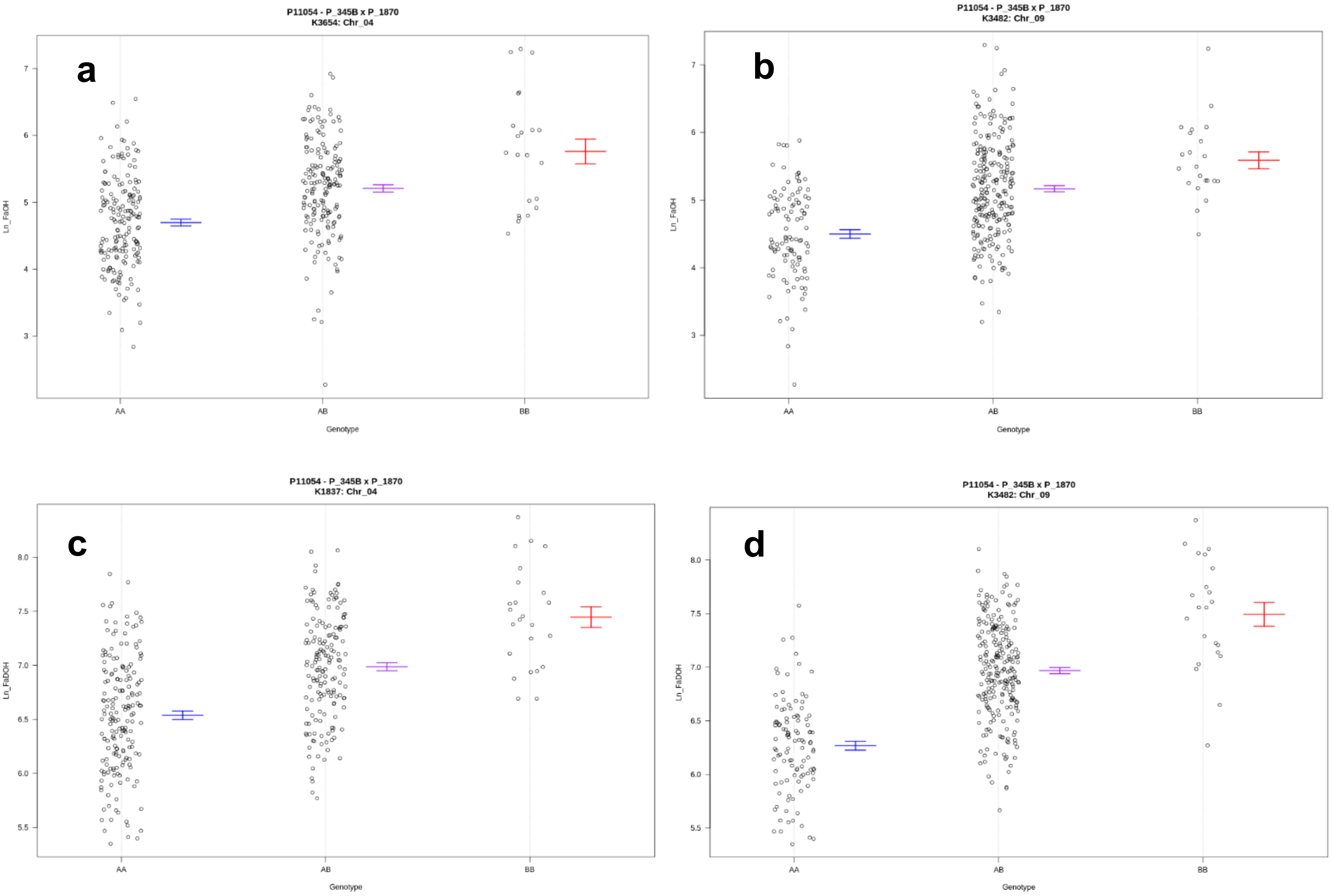

Figure S3 PA contents of marker genotypes. Phenotypic distribution is shown for peak markers of the following major QTLs: **a** *FaOH_4.1* (K3654), **b** *FaOH_9.1* (K3482), **c** *FaDOH_4.1* (K1837), **d** *FaDOH_9.1* (K3482).
