# Supplementary material for: The genetic control of polyacetylenes involved in bitterness of carrots (Daucus carota L.): Identification of QTLs and candidate genes from the plant fatty acid metabolism
Source: BMC Plant Biol. 2022 Mar 2;22:92. doi: 10.1186/s12870-022-03484-1 (PMC8889737; doi:10.1186/s12870-022-03484-1)
Supplement: Supplementary file 5 — Additional file 5: Data S1. Coding sequences (CDS) and predicted proteins of new FAD2s and CER1s. [file 12870_2022_3484_MOESM5_ESM.docx]

**Data S1:** Coding sequences (CDS) and predicted proteins of newly identified carrot *FAD2* and *CER1* genes.

***DcFAD2-25*** (chromosome 8)

**Coding sequence (CDS)**

ATGGGTGCAGGTGGACGCATGTCTGCTCCTAATGCCAAGAAAACTCAAACAGAAGCACTTCGACGTGCCCCTCATGAGAAACCTCCATTCACCATTGGTGACCTTAAGAAAGCCATTCCTGCTCATTGCTTTGAAAAGTCACTTGTCACTTCTTTTCGATACCTCATTCAAGATCTCCTCATGGCCTATGCCCTCTACTATATTGCCACTAATTACATAGAGCAGTATCTTCCATATCCTCTGAATTACGTCGGTTGGGCTGCTTACATTGCTGTCCAGGGGTGTGTCTTGACGGGAGCTTGGGTGGTGGGTCACGAATGTGATCACGACGCCTTCAGTGATTATGGTTGGGTGAATGACCTTGTTGGCCTTATTGTCCACTCTTCTCTCATGGTTCCATATTTCTCTTGGAAAATTAGCCACAGACGTCACCACGCCAACACTCAATCACTTGAGAACGACGAGGTTTATGTCCCGAGGTTCAAGTCCAACATCCGGAACTACTACAAAATTCTCAACAACCCACCCGGCCGTGTCCTTGTGTGGGTTACCACACTCCTCATAGGCTTCCCTCTGTATCTGATGTTCAATGTTTCGGGACACAAGTATGAGAGGTGGACTTCTCACTATGATCCCCATAGCCCTCTTTACACAGAACGTGAGCGCAAGCAGATCATTGTGTCTGATCTTGCCATTCTTGCTGTTATCTATGGGCTGTACAATCTAGTATTAGCCAAAGGATTTGTTTGGGTTTTCTGTGTTTATGGAGGTCCATTGCTAGTTGTCAACGGATGGTTTACATTAATTACAATCCTCAATCACACTCATCCTTCGATTCCTTACTACGACTCAACTGAATGGGACTGGTTAAGAGGAGCTCTCTGCACTGTTGACAGAGATTATGGAATTCTGAACAAGGTATTCCACAACGTGTGCAATGCTCATGTCTGTCACCACATATTCTCCATGATCCCACATTACCACGGACTAGAAGCCACAGAGGCCATGAAGCCTTTACTTGGAGATTACTATCAATATGATGGAACTCCAATTCTAAAGGCCATGTACCGAGAAATGAAGGAATGCATTTACGTGGAGAAAGATGAAGGCGAGACTAAAGGAGTCTACTGGTACCGAAAGGATATATAG

**Predicted protein**

MGAGGRMSAPNAKKTQTEALRRAPHEKPPFTIGDLKKAIPAHCFEKSLVTSFRYLIQDLLMAYALYYIATNYIEQYLPYPLNYVGWAAYIAVQGCVLTGAWVVGHECDHDAFSDYGWVNDLVGLIVHSSLMVPYFSWKISHRRHHANTQSLENDEVYVPRFKSNIRNYYKILNNPPGRVLVWVTTLLIGFPLYLMFNVSGHKYERWTSHYDPHSPLYTERERKQIIVSDLAILAVIYGLYNLVLAKGFVWVFCVYGGPLLVVNGWFTLITILNHTHPSIPYYDSTEWDWLRGALCTVDRDYGILNKVFHNVCNAHVCHHIFSMIPHYHGLEATEAMKPLLGDYYQYDGTPILKAMYREMKECIYVEKDEGETKGVYWYRKDI.

***DcFAD2-26*** (chromosome 8)

**Coding sequence (CDS)**

ATGGGTGCAGGTGGGCGAATGTCGGATCCTCCTAAAGGCAAAAAAACTGAAACAGAAGCACTTCGACGTGCTCCTCATGAGAAACCCCCTTTCACCATAGGTGACCTCAAGAAAGCCATTCCTGCTCATTGCTTCGAAAAATCACTCATCACTTCTTTCCGATATCTTATTCAAGATCTCCTCATGGCCTATGCCCTTTACTATGTTGCCACAAATTACATAGACCAGTATTTACCCCATCCAATCAACTACTTGGGTTGGGCAGTTTACATTGCTGTACAGGGCTGTGTCCTCACCGGGGCTTGGGTTGTAGGCCATGAGTGTGATCATGATGCCTTCAGTGACTATGGCTGGATCAATGACCTTGTTGGCCTTATTGTCCACTCTTCTCTCATGGTCCCTTATTTCTCTTGGAAAATTAGCCACAGACGTCACCACGCCAACACTCAGTCGCTTGAGAATGATGAAGTTTACGTTCCCAGATTCAAGTCCAACATCAGGAACTACTACAAAATTCTCAACAACCCACCCGGTCGTGTCCTTGTCTGGGTTACCACACTTCTCATAGGTTTCCCTCTATATTTGATGTTCAATGTTTCTGGACACAAGTATGAGAGGTGGACTTCACATTATGATCCCCACAGCCCTCTTTACACAGAACGTGAGCGCAAGCAGATCATTGTGTCTGATCTTGCCATTCTTGCTGTTATCTATGGGCTGTACAATCTAGTATTAGCCAAAGGATTTGTCTGGGTTTTCTGTGTCTATGGAGGTCCATTGCTTGTTGTCAATGGATGGTTCACATTGATCACCATCCTTAACCATACTCATCCCTCAGTGCCTTACTACGATTCAACCGAATGGGATTGGTTGAGGGGAGCTCTCTGCACTGTGGACAGAGATTATGGAATTTTGAACAAGGTATTCCACAATGTGTGCAATGCTCATGTCTGTCACCACATATTCTCCATGATCCCACATTACCACGGACTTGAAGCAACAGAGGCCATGAAGCCAGTGTTGGGCGATTATTATCAGTATGATGGAACTCCAATACTTAAGGCCATGTACAGAGAAATGAAGGAATGCATTTACGTGGAGAAGGATGAAGGCGAGACCAAAGGAGTCTACTGGTACAGAAAGGATATTTAG

**Predicted protein**

MGAGGRMSDPPKGKKTETEALRRAPHEKPPFTIGDLKKAIPAHCFEKSLITSFRYLIQDLLMAYALYYVATNYIDQYLPHPINYLGWAVYIAVQGCVLTGAWVVGHECDHDAFSDYGWINDLVGLIVHSSLMVPYFSWKISHRRHHANTQSLENDEVYVPRFKSNIRNYYKILNNPPGRVLVWVTTLLIGFPLYLMFNVSGHKYERWTSHYDPHSPLYTERERKQIIVSDLAILAVIYGLYNLVLAKGFVWVFCVYGGPLLVVNGWFTLITILNHTHPSVPYYDSTEWDWLRGALCTVDRDYGILNKVFHNVCNAHVCHHIFSMIPHYHGLEATEAMKPVLGDYYQYDGTPILKAMYREMKECIYVEKDEGETKGVYWYRKDI.

***DcFAD2-27*** (chromosome 8)

**Coding sequence (CDS)**

ATGGGTGCAGGTGGGCGTATGTCTGCTCCCTCTAGTGTCAAAAAAACTGAAACAGAGGCACTTCGACGTGCTCCTCATGAGAAACCTCCTTTCACCATTGGTGACCTCAAGAAAGCCATTCCTGCCCATTGCTTTGAAAAATCTTTAGTCACTTCTTTTCGATATCTCATTCAGGATCTTCTGATGGCCTATGCCCTCTACTATGTCGCTACTAATTACATCGACCAATATCTTCCTTATCCAATCAACTACTTGGGTTGGGCAGCTTATATTGCTGTTCAAGGCTGTGTGTTAACAGGGGCCTGGGTTGTGGGTCATGAATGTGATCATGATGCATTCAGCAATTATAATTGGATTAATGATCTCGTTGGCCTTGTTGTCCATTCTTCTCTCTTGGTGCCATACTTCTCTTGGAAAATTAGTCACCGTCGTCACCATGCCAATACTCAATCCCTTGAAAATGATGAGGTGTATGTCCCTAGATTCAAGTCCAACATCCGGAACTACTACAAAATTCTCAACAATCCACCAGGCCGTGTCCTCGTCTGGCTTATCACACTCCTCATAGGCTTCCCAATATACTTGATGTTCAATGTTTCGGGACACAAGTATGAGAGGTGGACTTCACACTATGATCCCCATAGCCCTCTTTACACAGAACGTGAGCGCAAGCAGATCATTGTGTCTGATCTTGCCATTCTTGCTGTTATCTATGGGCTGTACAATCTAGTATTAGCCAAAGGATTTGTCTGGGTTTTCTGTGTTTATGGAGGTCCGTTGCTCGTTGTCAACGGATGGTTCACATTGATCACCATCCTCAACCACACTCATCCCTCTGTGCCTTACTATGATTCAACCGAATGGGATTGGTTGAGGGGAGCTCTCTGCACTGTGGACAGAGATTATGGAATTTTGAACAAGGTATTCCACAATGTGTGCAATGCTCATGTGTGTCACCACATATTCTCCATGATCCCACATTACCACGGACTCGAAGCCACAGAGGCCATGAAGCCTGTATTGGGTGAATATTATCAGTATGATGGAACTCCGATTCTCAAGGCAATGTACAGAGAAATGAAGGAATGCATTTACGTGGAGAAAGATGAAGGCGAGACCAAAGGAGTCTACTGGTACAAAAAGGATATTTAG

**Predicted protein**

MGAGGRMSAPSSVKKTETEALRRAPHEKPPFTIGDLKKAIPAHCFEKSLVTSFRYLIQDLLMAYALYYVATNYIDQYLPYPINYLGWAAYIAVQGCVLTGAWVVGHECDHDAFSNYNWINDLVGLVVHSSLLVPYFSWKISHRRHHANTQSLENDEVYVPRFKSNIRNYYKILNNPPGRVLVWLITLLIGFPIYLMFNVSGHKYERWTSHYDPHSPLYTERERKQIIVSDLAILAVIYGLYNLVLAKGFVWVFCVYGGPLLVVNGWFTLITILNHTHPSVPYYDSTEWDWLRGALCTVDRDYGILNKVFHNVCNAHVCHHIFSMIPHYHGLEATEAMKPVLGEYYQYDGTPILKAMYREMKECIYVEKDEGETKGVYWYKKDI.

***DcFAD2-28*** (chromosome 8)

**Coding sequence (CDS)**

ATGGGTGCTGGTGGACGTATGTCCGCTCCTACTGGAAAGAAAACTGAAGCAGAAGCACTCCGACGTGCTCCTCATGAGAAACCTCCATTCACTATCGGAGACCTGAAAAAGGCTATTCCTGCCCATTGCTTTGAAAAATCAGTCATCACTTCTTTCCGTTACCTCATCCAGGATCTCATCATGGCCTATGCCCTCTACTATGTTGCCACAAACTATATAGACCAGTACTTACCCCATCCTCTTAACTACTTGGGTTGGGCAGCTTACATTGCTGTTCAGGGCTGTGTCTTGACCGGGGCTTGGGTTGTAGGCCATGAATGTGATCATGATGCCTTCAGTGATTATGGTTGGGTCAATGACCTTGTTGGCCTTGTTGTCCATTCTTCTCTTCTGGTCCCTTACTTCTCTTGGAAAATTAGCCACCGTCGTCATCATGCCAACACTCAGTCCCTTGAGAATGATGAGGTTTATGTCCCAAGATTCAAATCCAACATCAGGAACTACTACAAAATCATGAACAACCCACCCGGTCGTGTCCTTGTCTGGCTTATCACACTCCTCATAGGCTTCCCTTTATACTTGATGTTCAACGTCTCTGGACACAAGTATGAGAGGTGGACTTCACACTACGATCCCCACAGCCCTCTTTACACTGAACGCGAGCGCAAGCAGATCATTGTTTCTGATGTTGCAATCCTTGCTGTTATCTATGGCTTATACAATCTAGTACTAGCCAAGGGATTTGTCTGGGTTTTCTGTGTTTATGGAGGTCCATTGCTCGTCGTTAATGGATGGTTCACATTGATCACCATCCTCAACCATACTCATCCTTCTTTGCCTTACTATGATTCAACCGAATGGGATTGGTTGAGAGGAGCTCTTTGCACTGTGGACAGAGATTATGGAATTTTGAACAAGGTGTTCCACAATGTCTGTAATGCTCATGTTTGTCACCATATCTTCTCCATGATCCCACATTACCATGGACTCGAAGCAACCGAGGCAATGAAGCCAGTACTGGGAAACTATTATCAGTATGATGGGACTCCAATTCTCAAGGCAATGTACAGAGAAATGAAGGAATGCATTTATGTCCAAAAGGATGAAGGTGAGACCAAAGGAGTCTACTGGTACAGAAAGGAATTTTAG

**Predicted protein**

MGAGGRMSAPTGKKTEAEALRRAPHEKPPFTIGDLKKAIPAHCFEKSVITSFRYLIQDLIMAYALYYVATNYIDQYLPHPLNYLGWAAYIAVQGCVLTGAWVVGHECDHDAFSDYGWVNDLVGLVVHSSLLVPYFSWKISHRRHHANTQSLENDEVYVPRFKSNIRNYYKIMNNPPGRVLVWLITLLIGFPLYLMFNVSGHKYERWTSHYDPHSPLYTERERKQIIVSDVAILAVIYGLYNLVLAKGFVWVFCVYGGPLLVVNGWFTLITILNHTHPSLPYYDSTEWDWLRGALCTVDRDYGILNKVFHNVCNAHVCHHIFSMIPHYHGLEATEAMKPVLGNYYQYDGTPILKAMYREMKECIYVQKDEGETKGVYWYRKEF.

***DcFAD2-29*** (chromosome 1)

**Coding sequence (CDS)**

ATGGATCAGATGTCGGATTCGGATAAAAAAGTATCAAGAGTTCCTGTCTCAAGACCCGAATTCACTATCAGTGACCTTAAGAAAGCTATACCTCCCCATTGCTTTAAGCGTTCTGCTTTTCGCTCTTTTTCATACCTTCTCTCTGATATCACCGTTTTCTTCTCTCTTTCGTACATTTTCACAACAATCATGACACAACCTATGCATCAAATCTACTTCTATATGACCTGCATTGTTTATTCAGTGATACAAGGCTGTGTATTCTCACGGTTCTGGATCATAGGCCACGAATGTGGTCACAGTGCTTTCAGTGAGTACAAATGGCTAGACGACACAGTTGGATTCACCCTGCACTCCTTCCTCCTGTTTCCTTACTTCTCGTTTAAGTACAGTCACCACCGCCACCACACAAAAACCGGTTCTCTTGAACAAGACGAACTAGACATCCCTCTGCTCAAGTCCCAAGTTCCATCAATTCACAAGCACTTGACCACACATCCTGTACCAAGATTCTTGGTCATCACTCTGGTTTTAATCTTCGGTGTGCCTCTATACTTGCTTGTAAACTTCCGTGGTCGTGCTTATAATCAATTCGCCTCTCATTTCTATCGGTTTAGTCCAATGTACTCACCTAACCAACGCGCTCAAATCTTACTTTCTGACACTGCATTACTCGCCATGCTCTATGGACTATACACACTCGTTTCACTAAAAGGATTTGCTTGGATTGCTCTTGTTTACGGAGGACCACACCTGGTTCATACTGGCATGCTTTTCGTCGTGGCCTTACTCCACCACACTCACCCTCTAGTACCTTACTATGATTCCACAGAGTGGGATTGGCTAAGGGGATCATTGTCTACCATTGATCGAAACTATGGAATCCTTGACACAATCTATTATCAAGCAACCAATACTCATGTAGCACATCATCTCTTCACATCAATCCCACATTACCATGCAATGGAGGCCACCAGAGCAATCAAACCGATACTCGGAGAGTACTACAGGTATGATGGCACAATATTTTACAAATCGCTGTGGAGTGCTATTAAAGAGTGTGTCTATGTCGAGGAAGATGAAAGCAAAGGGATTTATTGGTATAACAACAAGCCCTGA

**Predicted protein**

MDQMSDSDKKVSRVPVSRPEFTISDLKKAIPPHCFKRSAFRSFSYLLSDITVFFSLSYIFTTIMTQPMHQIYFYMTCIVYSVIQGCVFSRFWIIGHECGHSAFSEYKWLDDTVGFTLHSFLLFPYFSFKYSHHRHHTKTGSLEQDELDIPLLKSQVPSIHKHLTTHPVPRFLVITLVLIFGVPLYLLVNFRGRAYNQFASHFYRFSPMYSPNQRAQILLSDTALLAMLYGLYTLVSLKGFAWIALVYGGPHLVHTGMLFVVALLHHTHPLVPYYDSTEWDWLRGSLSTIDRNYGILDTIYYQATNTHVAHHLFTSIPHYHAMEATRAIKPILGEYYRYDGTIFYKSLWSAIKECVYVEEDESKGIYWYNNKP.

***DcFAD2-30*** (chromosome 3)

**Coding sequence (CDS)**

ATGGAGCGAAAAAATGTATCCAGAACTCCCCAGTTGAAACCTCCTTTCACTCTTGGTGATGTTAAGAAAGCAATTCCCCCTCATTGCTTTGAGCGTCCTGTTCTTCGCTCTTTCTCATTCCTTCTCTTTGATGTCGTCATGTTATGCTCCCTTTTCTGCATTTTCGCAAAATTTATCATTACAGAGCAGTCTCGGTTTTATTTCCTTGTGTATATGGTCATGTACTCACAGCTTCAAGGCTGTGTGTTCTCTCGGTTTTGGATCATAGGCCATGAATGTGGTCATAATGCTTTTAGTGACTACAGATGGCTGAATGACACAGTCGGATTCATCGTCCACTCCTTCCTCCTGTTTCCTTACTTCTCATGGAAGTACAATCACCGTCGTCACCACTCCAGAACAGGACATCTGCACAAAGAAGAATTCAACGGCCCAATGCTCAAGTCTGAGGTTCCCTTAATTTTCAAGCACCTGATCACGAATCCTGCAACAAGATTCTTGGTTACTTTCATAGTTTTAGCTTTTGGTGTGCCTCTATACTTGCTTGTCAACTTTCGTGGTCGAGCTTATGATCGATTTGCCTCCCAATTTGATCCCTATAGTCCCATGTTTTCACGTAACCAACGCGCTCAAGTCTTTGTTTCTGATGCTGCATTTCTGACCATGGTCTATGCACTGTACAAACTTGTTTCATTGAAAGGCTTTGCATGGGTTGCTTGTGCTTACGGAGGACCATACCTAGTTCAGACTTCTAATGTCTTCCTAGTAGCTATACTTCAACACACGCACCCTTTCGTACCTTTCTACGATTCCAGTGAGTGGGATTGGCTAAGGGGATCTCTGGGTACCATTGACAGAGATTTTGGAATTCTCAATACTATGCATTATCAATCAACAAATACTCATGTAGCTCACCATCTGTTCCCAACAATCCCACATTACCATGCAAAGGAAGCCACTGAAGCCATTAAGCCTATTCTGGGGGAATACTATCGGTATGATGACACCCCAATCTATAAAGCACTGTGGACTACTTTTAAGGAATGTGTTTATGTTGAGGAAGATGAAGGTGACCAAAACAAAGGGATATATTGGTATAAATATAAGTTCTGA

**Predicted protein**

MERKNVSRTPQLKPPFTLGDVKKAIPPHCFERPVLRSFSFLLFDVVMLCSLFCIFAKFIITEQSRFYFLVYMVMYSQLQGCVFSRFWIIGHECGHNAFSDYRWLNDTVGFIVHSFLLFPYFSWKYNHRRHHSRTGHLHKEEFNGPMLKSEVPLIFKHLITNPATRFLVTFIVLAFGVPLYLLVNFRGRAYDRFASQFDPYSPMFSRNQRAQVFVSDAAFLTMVYALYKLVSLKGFAWVACAYGGPYLVQTSNVFLVAILQHTHPFVPFYDSSEWDWLRGSLGTIDRDFGILNTMHYQSTNTHVAHHLFPTIPHYHAKEATEAIKPILGEYYRYDDTPIYKALWTTFKECVYVEEDEGDQNKGIYWYKYKF.

***DcFAD2-31*** (chromosome 4)

**Coding sequence (CDS)**

ATGGACCGAAAATCGGATTGTAGCGTGAAAAGAACATTAAGATCTCCCCACACCAAACCTCCATTCACTCTAAGTGATGTCAAGAAAGCAGTTCCACCACATTGTTTTCAGCGTTCGGCTATTCGCTCTTTGTCATATCTCGCCTTAGATCTCTTCATTTCTTTCTCTCTTTACTACATTGCCGCAACCTATATCTTAACATCGGAAGTAGTCAAGTCATCCAAATTATATTTTCTTACTTGTTGGATTATATATTCATTGCTTCAAGGATGCATACTTGCTCGATTTTGGGTCATAGGTCACGAATGTGGGCACGGGGCATTCAGCGACTACAAATGGCTCGACGATACAGTCGGATTCCTCACACACTCTCTCGTCCTGTTTCCTTATTTTTCATTCAAGTTTAGTCACCATCGTCACCACTTGAGAACCGGATCCCTCGAAGAGGAGGAATTCGACATCCCTTTGCTCAAGAATCAAGTCTCGTTTATTTTTAAATACCTCAACAATCCTGTCGCCAGATTTTTTGTCATTCTCTTGGTTCTAATTGTTGCGGTGCCTCTGTACTTGCTTGTCAACTTCCGTGGCCGTACTTACGAC**TGA**TTTGCCTCTCATTTTGATCCGTATAGTCCAATGTTCTCACGAAAACAACGTGCTCAAGTCTTGCTTTCGGATGCTGGATGTTTGGCCGTGATCTATGCAGTCTACAAACTTGCTCTGTTGAAAGGCTTCGCTTGGATTGGTTTTATTTACGGAGGACCGTACCTGTTCCAAAATGCAATGCTGATCATAGTGGCTGTACTTCAACACACAAACCCTCTTGTGCCTTACTACAATTCCACAGAGTGGGAGTGGTTGAAGGGATCCATGGCCACTATTGATCGCGATTTTGGATTTCTTAACACGGTGTTTCATCAGCAGCCGAATACACATGTAGCGCATCATCTCTTTCCCAAAATGCCGCATTACCATGAAGTGGAAGCCACCAGAGCATTTAAACCTATATTGGGGGAGTACTATCAGTTTGATTACACACCATTTTATAAATCACTGTGGAGTACTCTAAAAGATTGTGTATATGTTGAGGAAGATGAGCAAAACAAAGGGATTTATTGGTATAACAATAAGTTTTAA

**Predicted protein**

MDRKSDCSVKRTLRSPHTKPPFTLSDVKKAVPPHCFQRSAIRSLSYLALDLFISFSLYYIAATYILTSEVVKSSKLYFLTCWIIYSLLQGCILARFWVIGHECGHGAFSDYKWLDDTVGFLTHSLVLFPYFSFKFSHHRHHLRTGSLEEEEFDIPLLKNQVSFIFKYLNNPVARFFVILLVLIVAVPLYLLVNFRGRTYD**.**FASHFDPYSPMFSRKQRAQVLLSDAGCLAVIYAVYKLALLKGFAWIGFIYGGPYLFQNAMLIIVAVLQHTNPLVPYYNSTEWEWLKGSMATIDRDFGFLNTVFHQQPNTHVAHHLFPKMPHYHEVEATRAFKPILGEYYQFDYTPFYKSLWSTLKDCVYVEEDEQNKGIYWYNNKF.

*Note the premature stop codon at position nt 601-603 (sequence not yet verified by cloning and resequencing)*

***DcCER1-3*** (chromosome 7)

**Coding sequence (CDS)**

ATGGCTACTAACCCGGGCATTCTCACTGACTGGCCATGGACACCCCTTGGAAGCTACAAGTATGTGGTTTTGGCACCATTTGTGGTTCATAGCATTCACTCGTACATAACGAAGGATGAGAACGAAAGAGACTTGTCCAACTTTCTGATATTCCCGTTTCTTCTGTGGAGGATGCTTCATAATCAGATTTGGATTTCCCTTTCTCGCTACAGAACTGCCAAAGGCAACAACCGGATTGTTGATAGAACTATTGAATTTGAGCAGGTTGATCGAGAGAGAAACTGGGATGACCAAATCTTGTTTAATGGGATACTGTATTATTTGGTTAACTTGACAATGAAAGGAGGGTCTCATCTGCCTTTTTGGAGGACTGATGGTATTCTTATCACAATTCTGATTCATGCTGGACCTGTTGAATTCATCTACTACTGGCTTCACAGAGCATTACACCACCACTACCTCTATTCTCGCTATCATTCTCATCATCATTCCTCTATTGTTACTGAGCCTATTACATCTGTTATTCATCCATTTGCAGAACACATATCGTATTTTTTGCTCTTTGCAATACCACTGATGACCGCTGCACTGACGGAGACGGGCTCTATCGCTTTATTCCTTTTTTACATAACTTTTGTGGACTTCATGAACAATATGGGACACTGCAACTTTGAGCTTATCCCTAAGAAGCTGTTTTCCATCTTTCCTCCTCTCAAGTACATCATGTATACGCCCTCGTACCATTCTCTGCATCATACACAATTCCGAACCAACTACTCGCTTTTCATGCCTTTCTATGACTACATGTATGGCACCATGGACAAGTCTACAGACACATTATATGAAACTTCACTCAAAAGAGAGGAGGAGTCTGCTAATGTGGTACATCTGACACATCTGACCACACCAGAATCTATTTATCATCTTCGTGTTGGATTCGCCTCTTTAGCATCAAAACCCCAGAGTACTTCACAGTGGTACATGTGGTTAATGTGGCCTGTGACATTCTGGTCTATGATTGTTACATGGTTTTACGGTCAAACATTTGTTATTGAGAGGAATATATTCAAAAATCTGAACTTGCAAACATGGGCTATTCCAAGATACAGCATACAATACACTACAGTCAAGCAAAGAGAGAGTATCAATTGTTTGATTGAAGAGGCGATAGTTGAAGCTGAGAGAAAAGGAATTATGGTTTTAACTTTAGGCCTTCTTAATCAGGGAGAGGAGATGAACAGCAATGGTGAGCTTTTTATACGAAGGAATCCTAAGCTAAAAGTGAAGTTAGTTGATGGGAGTAGCCTAGCAGTTGCTGTTGTTCTGAATAGCATTCCAAAGGGAACGACTCAAGTCGCCATTAAAGGCAACTTATCCAAGGTCTCCAATTCCATTGCCATTGCTTTGTGTCGCAGAGGTGTCCAGGTATTTATTTCCTGCGAATATGGTTATAAAAGGCTCACGGAAACGTGTGATTCTGAGACTCAAAAGAATTTGGTCCTTTCAGATAGTTCTTCTCAGCAGATATGGTTAGTGGGAGATAAATTGGGAAAGAAAGAACAGATGAAGGCCTCAAAGGGAACATTATTCATCCCTTTCTCTCAGTTTCCCCCAAAGAAATTGCGCAAAGATTGCTTCTACTGCAATATTCCAGCAATGTCTGCTCCAGTGCATCTCCAGAATCTGGACTCTTGTGAGAATTGGTTGCCAAGAAGGGTGATGAGTGCATGGCGTATAGCTGGAATAGTGCATGCATTAGAAGAATGGAATGTACATGAATGTGGCAATATGATGTTCAGCATCGAAAAGATTTGGAAAGCTACTCTTGAGCATGGGTTTCGTCCCCTCCCAGTGCCCACATAA

**Predicted protein**

MATNPGILTDWPWTPLGSYKYVVLAPFVVHSIHSYITKDENERDLSNFLIFPFLLWRMLHNQIWISLSRYRTAKGNNRIVDRTIEFEQVDRERNWDDQILFNGILYYLVNLTMKGGSHLPFWRTDGILITILIHAGPVEFIYYWLHRALHHHYLYSRYHSHHHSSIVTEPITSVIHPFAEHISYFLLFAIPLMTAALTETGSIALFLFYITFVDFMNNMGHCNFELIPKKLFSIFPPLKYIMYTPSYHSLHHTQFRTNYSLFMPFYDYMYGTMDKSTDTLYETSLKREEESANVVHLTHLTTPESIYHLRVGFASLASKPQSTSQWYMWLMWPVTFWSMIVTWFYGQTFVIERNIFKNLNLQTWAIPRYSIQYTTVKQRESINCLIEEAIVEAERKGIMVLTLGLLNQGEEMNSNGELFIRRNPKLKVKLVDGSSLAVAVVLNSIPKGTTQVAIKGNLSKVSNSIAIALCRRGVQVFISCEYGYKRLTETCDSETQKNLVLSDSSSQQIWLVGDKLGKKEQMKASKGTLFIPFSQFPPKKLRKDCFYCNIPAMSAPVHLQNLDSCENWLPRRVMSAWRIAGIVHALEEWNVHECGNMMFSIEKIWKATLEHGFRPLPVPT.
