# Supplementary material for: The genetic control of polyacetylenes involved in bitterness of carrots (Daucus carota L.): Identification of QTLs and candidate genes from the plant fatty acid metabolism
Source: BMC Plant Biol. 2022 Mar 2;22:92. doi: 10.1186/s12870-022-03484-1 (PMC8889737; doi:10.1186/s12870-022-03484-1)
Supplement: Supplementary file 6 — Additional file 6: Table S2. List of FAD2 gene models and their genomic positions. [file 12870_2022_3484_MOESM6_ESM.docx]

**Table S2** List of 31 *Daucus carota* fatty acid desaturase (*FAD2*) gene models sorted by their physical position on the assembled nine carrot chromosomes according to the whole genome sequence [34] and the published carrot *FAD2* inventory [5].

Genomic position^1)^  CDS No. Protein

| Chromosome^1)^ | Gene name | Strand | Start | Stop | Locus name^2)^ | Proved function^3)^ | length | Introns | lenght |
| --- | --- | --- | --- | --- | --- | --- | --- | --- | --- |
| 1 | *DcFAD2-29* | for | 2623973 | 2625091 | n.a. |  | 1119 | 0 | 373 |
| 1 | *DcFAD2-18* | for | 24320363 | 24321512 | DCAR_002026 |  | 1149 | 0 | 382 |
| 1 | *DcFAD2-3* | for | 38720179 | 38721331 | DCAR_003420 |  | 1152 | 0 | 383 |
| 3 | *DcFAD2-30* | rev | 11882922 | 11884034 | n.a. |  | 1113 | 0 | 371 |
| 3 | *DcFAD2-14* | for | 39497199 | 39498366 | DCAR_011708 |  | 1167 | 0 | 388 |
| 3 | *DcFAD2-15* | rev | 39502342 | 39503509 | DCAR_011709 |  | 1167 | 0 | 388 |
| 4 | *DcFAD2-31* | rev | 10151245 | 10152378 | n.a. |  | 1134 | 0 | 378 |
| 4 | *DcFAD2-22* | for | 29308974 | 29310153 | DCAR_013553 |  | 1179 | 0 | 392 |
| 4 | *DcFAD2-7* | for | 29312792 | 29313944 | DCAR_013552 | delta12 acetylenase | 1152 | 0 | 383 |
| 4 | *DcFAD2-17* | for | 29318510 | 29319686 | DCAR_013551 |  | 1176 | 0 | 391 |
| 4 | *DcFAD2-16* | rev | 29340355 | 29341522 | DCAR_013549 |  | 1167 | 0 | 388 |
| 4 | *DcFAD2-8* | rev | 29343013 | 29344165 | DCAR_013548 | delta12 acetylenase | 1152 | 0 | 383 |
| 4 | *DcFAD2-19* | for | 29348356 | 29349505 | DCAR_013547 | bifunct. desaturase | 1140 | 0 | 382 |
| 5 | *DcFAD2-13* | rev | 10549274 | 10550426 | DCAR_017010 |  | 1152 | 0 | 383 |
| 5 | *DcFAD2-6* | rev | 10559828 | 10560980 | DCAR_017011 | delta12 acetylenase | 1152 | 0 | 383 |
| 5 | *DcFAD2-23* | rev | 10593543 | 10594722 | DCAR_017012 |  | 1179 | 0 | 392 |
| 5 | *DcFAD2-24* | for | 24911311 | 24912904 | DCAR_017923 |  | 1104 | 1? | 367 |
| 5 | *DcFAD2-11* | rev | 41732457 | 41733609 | DCAR_019786 | bifunct. desaturase | 1152 | 0 | 383 |
| 5 | *DcFAD2-20* | rev | 41738175 | 41739333 | DCAR_019787 |  | 1158 | 0 | 385 |
| 6 | *DcFAD2-10* | rev | 33657256 | 33658405 | DCAR_020161 |  | 1149 | 0 | 382 |
| 6 | *DcFAD2-12* | rev | 36316385 | 36317537 | DCAR_019845 |  | 1152 | 0 | 383 |
| 7 | *DcFAD2-5* | rev | 32083676 | 32084828 | DCAR_025967 |  | 1152 | 0 | 383 |
| 8 | *DcFAD2-4* | rev | 20875213 | 20876365 | DCAR_027655 |  | 1152 | 0 | 383 |
| 8 | *DcFAD2-25* | rev | 20877925 | 20879073 | n.a. |  | 1149 | 0 | 383 |
| 8 | *DcFAD2-26* | rev | 20881526 | 20882677 | n.a. |  | 1152 | 0 | 383 |
| 8 | *DcFAD2-27* | rev | 20885064 | 20886215 | n.a. |  | 1152 | 0 | 383 |
| 8 | *DcFAD2-28* | for | 20887620 | 20888768 | n.a. |  | 1149 | 0 | 383 |
| 8 | *DcFAD2-1* | rev | 21164452 | 21165604 | DCAR_027616 |  | 1152 | 0 | 383 |
| 8 | *DcFAD2-9* | rev | 21168523 | 21169678 | DCAR_027615 |  | 1155 | 0 | 384 |
| 8 | *DcFAD2-21* | for | 21171805 | 21172963 | DCAR_027614 |  | 1158 | 0 | 385 |
| 8 | *DcFAD2-2* | rev | 21538656 | 21539808 | DCAR_027583 |  | 1152 | 0 | 383 |

| ^1)^ Chromosomes, genomic coordinates, orientation and locus names according the carrot whole genome sequence assembly vers.2 [34] | |
| --- | --- |
| ^2)^ DCAR locus identified as *FAD2* gene by Busta et al. [5]; n.a. - not annotated in carrot genome vers.2 (this study)  ^3)^ according Busta et al. [5] | |
|  |  |
