# Supplementary material for: The genetic control of polyacetylenes involved in bitterness of carrots (Daucus carota L.): Identification of QTLs and candidate genes from the plant fatty acid metabolism
Source: BMC Plant Biol. 2022 Mar 2;22:92. doi: 10.1186/s12870-022-03484-1 (PMC8889737; doi:10.1186/s12870-022-03484-1)
Supplement: Supplementary file 7 — Additional file 7: Figure S4. Alignment of predicted carrot FAD2 protein sequences. [file 12870_2022_3484_MOESM7_ESM.pdf]

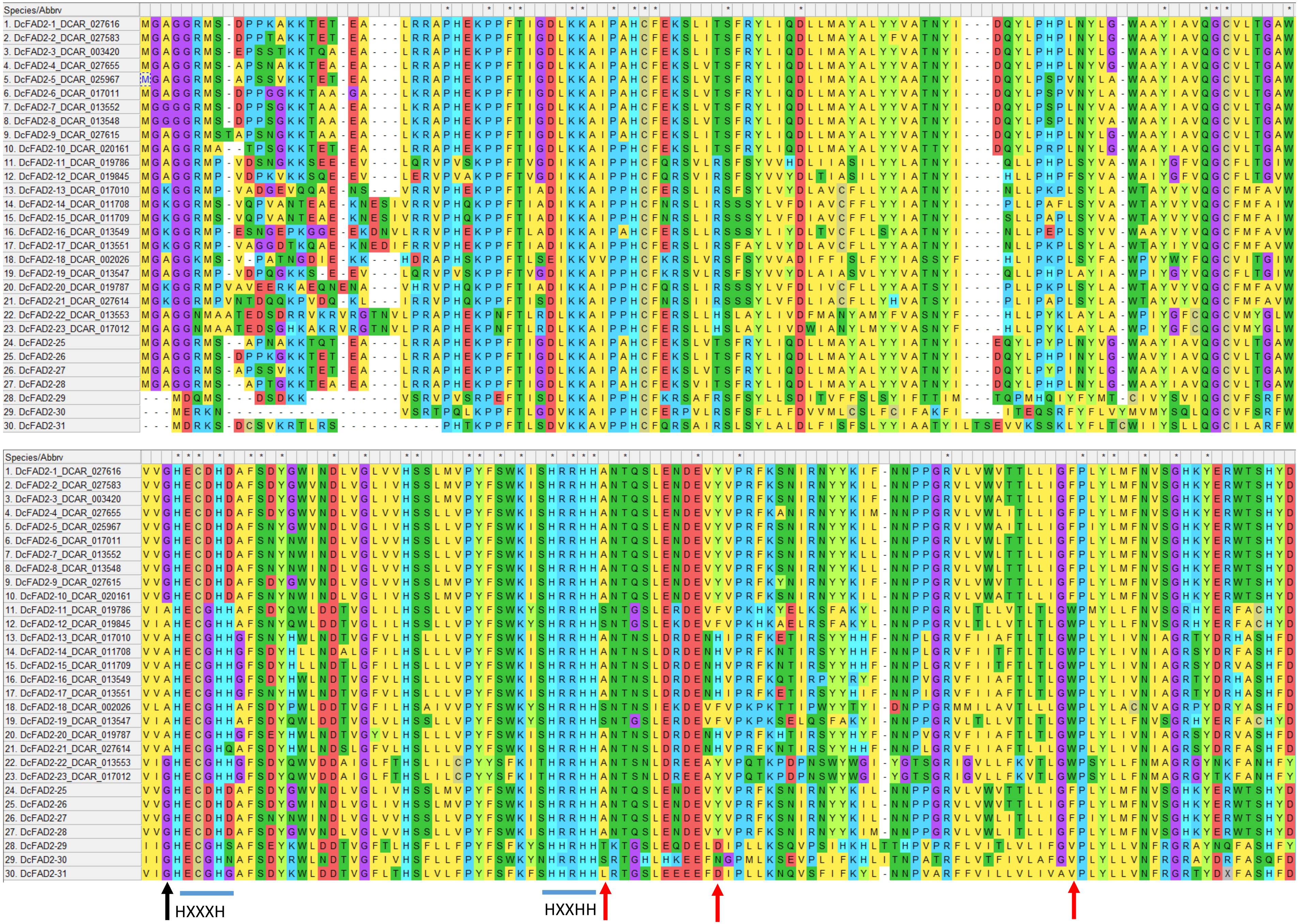
Species/Abbrv
1. DcFAD2-1_DCAR_027616
2. DcFAD2-2_DCAR_027583
3. DcFAD2-3_DCAR_003420
4. DcFAD2-4_DCAR_027655
5. DcFAD2-5_DCAR_025967
6. DcFAD2-6_DCAR_017011
7. DcFAD2-7_DCAR_013552
8. DcFAD2-8_DCAR_013548
9. DcFAD2-9_DCAR_027615
10. DcFAD2-10_DCAR_020161
11. DcFAD2-11_DCAR_019786
12. DcFAD2-12_DCAR_019845
13. DcFAD2-13_DCAR_017010
14. DcFAD2-14_DCAR_011708
15. DcFAD2-15_DCAR_011709
16. DcFAD2-16_DCAR_013549
17. DcFAD2-17_DCAR_013551
18. DcFAD2-18_DCAR_002026
19. DcFAD2-19_DCAR_013547
20. DcFAD2-20_DCAR_019787
21. DcFAD2-21_DCAR_027614
22. DcFAD2-22_DCAR_013553
23. DcFAD2-23_DCAR_017012
24. DcFAD2-25
25. DcFAD2-26
26. DcFAD2-27
27. DcFAD2-28
28. DcFAD2-29
29. DcFAD2-30
30. DcFAD2-31
HXXXH
HXXHH

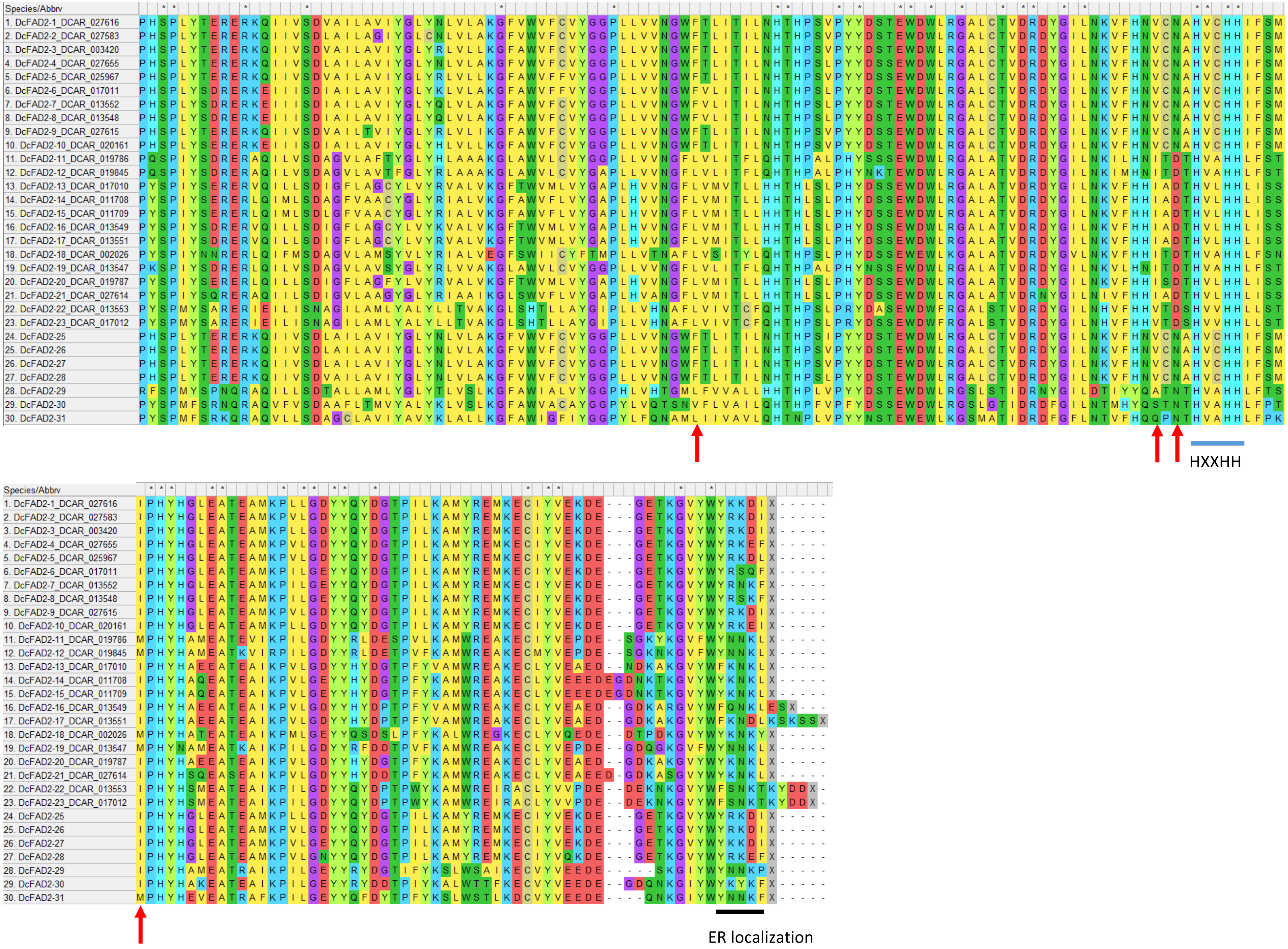
Species/Abbrv
1. DcFAD2-1_DCAR_027616
2. DcFAD2-2_DCAR_027583
3. DcFAD2-3_DCAR_003420
4. DcFAD2-4_DCAR_027655
5. DcFAD2-5_DCAR_025967
6. DcFAD2-6_DCAR_017011
7. DcFAD2-7_DCAR_013552
8. DcFAD2-8_DCAR_013548
9. DcFAD2-9_DCAR_027615
10. DcFAD2-10_DCAR_020161
11. DcFAD2-11_DCAR_019786
12. DcFAD2-12_DCAR_019845
13. DcFAD2-13_DCAR_017010
14. DcFAD2-14_DCAR_011708
15. DcFAD2-15_DCAR_011709
16. DcFAD2-16_DCAR_013549
17. DcFAD2-17_DCAR_013551
18. DcFAD2-18_DCAR_002026
19. DcFAD2-19_DCAR_013547
20. DcFAD2-20_DCAR_019787
21. DcFAD2-21_DCAR_027614
22. DcFAD2-22_DCAR_013553
23. DcFAD2-23_DCAR_017012
24. DcFAD2-25
25. DcFAD2-26
26. DcFAD2-27
27. DcFAD2-28
28. DcFAD2-29
29. DcFAD2-30
30. DcFAD2-31
HXXHH
ER localization

Figure S4 Alignment (MEGA-X, Muscle) of the predicted protein sequences of 23 *D. carota FAD2s* [5] and seven newly identified carrot *FAD2s* (*DcFAD2-25* to *DcFAD2-31*, this study). Three putative His boxes are underlined in blue. The position of the G/A residues putatively promoting acetylenase activity is marked by a black arrow. Residues indicating divergent FAD2 function (according [5]) are marked with red arrows. The putative ER localization motif at the C-terminus is underlined in black.
