# Supplementary material for: The genetic control of polyacetylenes involved in bitterness of carrots (Daucus carota L.): Identification of QTLs and candidate genes from the plant fatty acid metabolism
Source: BMC Plant Biol. 2022 Mar 2;22:92. doi: 10.1186/s12870-022-03484-1 (PMC8889737; doi:10.1186/s12870-022-03484-1)
Supplement: Supplementary file 8 — Additional file 8: Figure S5. Phylogenetic tree of carrot FAD2 protein sequences. [file 12870_2022_3484_MOESM8_ESM.pdf]

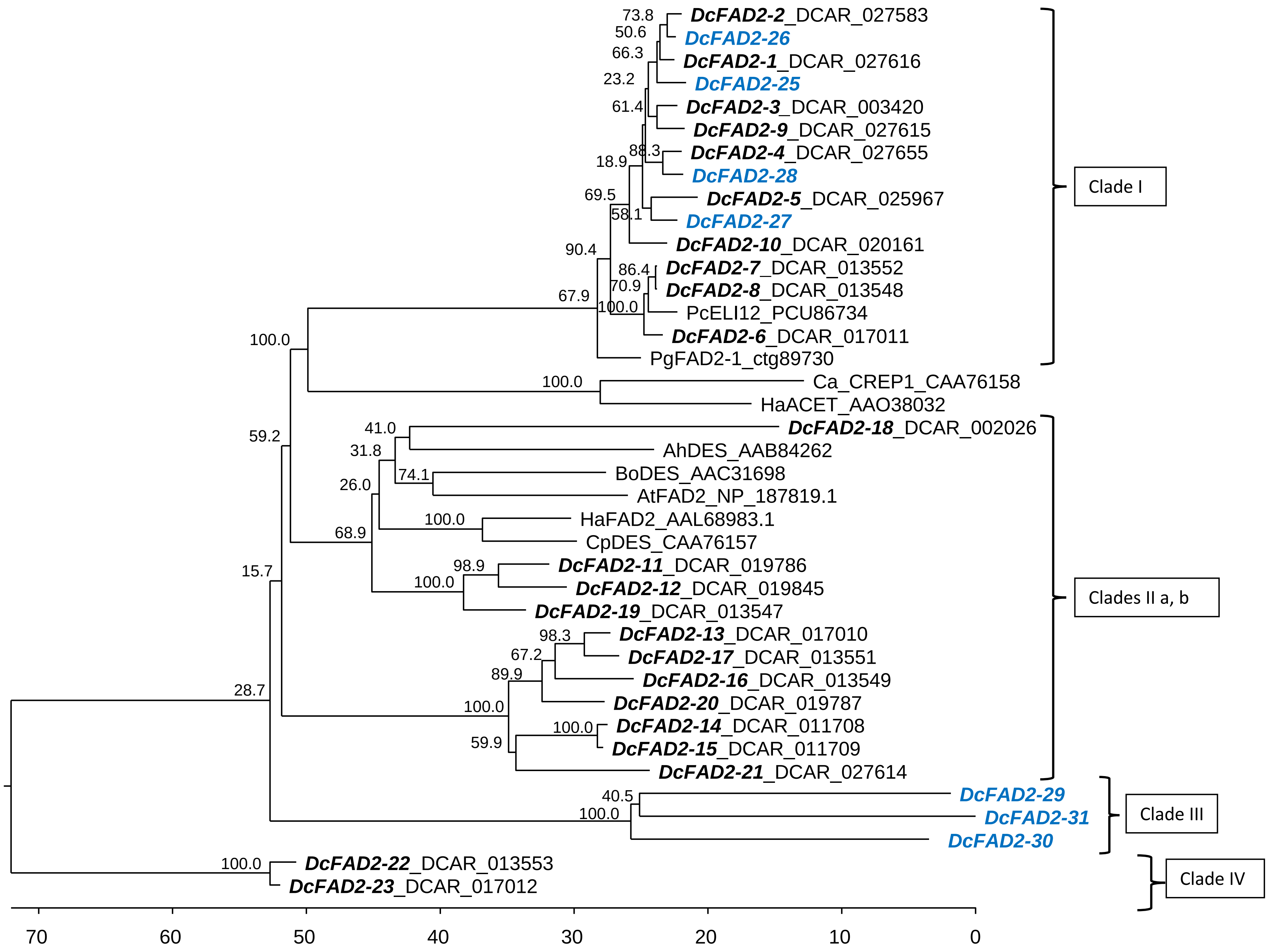
DcFAD2-2_DCAR_027583
DcFAD2-26
DcFAD2-1_DCAR_027616
DcFAD2-25
DcFAD2-3_DCAR_003420
DcFAD2-9_DCAR_027615
DcFAD2-4_DCAR_027655
DcFAD2-28
DcFAD2-5_DCAR_025967
DcFAD2-27
DcFAD2-10_DCAR_020161
DcFAD2-7_DCAR_013552
DcFAD2-8_DCAR_013548
PcELI12_PCU86734
DcFAD2-6_DCAR_017011
PgFAD2-1_ctg89730
Ca_CREP1_CAA76158
HaACET_AAO38032
DcFAD2-18_DCAR_002026
AhDES_AAB84262
BoDES_AAC31698
AtFAD2_NP_187819.1
HaFAD2_AAL68983.1
CpDES_CAA76157
DcFAD2-11_DCAR_019786
DcFAD2-12_DCAR_019845
DcFAD2-19_DCAR_013547
DcFAD2-13_DCAR_017010
DcFAD2-17_DCAR_013551
DcFAD2-16_DCAR_013549
DcFAD2-20_DCAR_019787
DcFAD2-14_DCAR_011708
DcFAD2-15_DCAR_011709
DcFAD2-21_DCAR_027614
DcFAD2-29
DcFAD2-31
DcFAD2-30
DcFAD2-22_DCAR_013553
DcFAD2-23_DCAR_017012
Clade I
Clades II a, b
Clade III
Clade IV
73.8
50.6
66.3
23.2
61.4
88.3
18.9
69.5
58.1
90.4
86.4
70.9
67.9
100.0
100.0
100.0
59.2
41.0
31.8
74.1
26.0
100.0
68.9
98.9
100.0
15.7
98.3
67.2
89.9
28.7
100.0
100.0
59.9
40.5
100.0
100.0
70
60
50
40
30
20
10
0

Figure S5 Phylogenetic tree of *Daucus FAD2* proteins (DCAR numbers, [5]), the published *Petroselinum* ELI12 acetylenase [36], a *Panax ginseng FAD2* and some other putative acetylenases and desaturases (numbers are NCBI accession numbers; Dc, *Daucus carota*; Pc, *Petroselinum crispum*; Pg, *Panax ginseng*; Ca, *Crepis alpina*; Ha, *Helianthus annuus;* At, *Arabidopsis thaliana*; Bo, *Borago officinalis*; Cp, *Crepis palaestina*; Ah, *Arachis hypogaea*). Newly identified carrot *FAD2s* are labeled in blue (this study). Multiple sequence alignment was performed by ClustalW using the Lasergene (DNASTAR) software package. A phylogenetic tree was constructed using the Kimura distance formula to calculate distance values and bootstrap analysis (1,000 replicates). Numbers indicate bootstrap replication, and branch length is scaled below the tree indicating the number of amino acid substitutions per 100 amino acids.
