# Supplementary material for: The genetic control of polyacetylenes involved in bitterness of carrots (Daucus carota L.): Identification of QTLs and candidate genes from the plant fatty acid metabolism
Source: BMC Plant Biol. 2022 Mar 2;22:92. doi: 10.1186/s12870-022-03484-1 (PMC8889737; doi:10.1186/s12870-022-03484-1)
Supplement: Supplementary file 9 — Additional file 9: Figure S6. Alignment of predicted carrot CER1 and CER3 protein sequences. [file 12870_2022_3484_MOESM9_ESM.pdf]

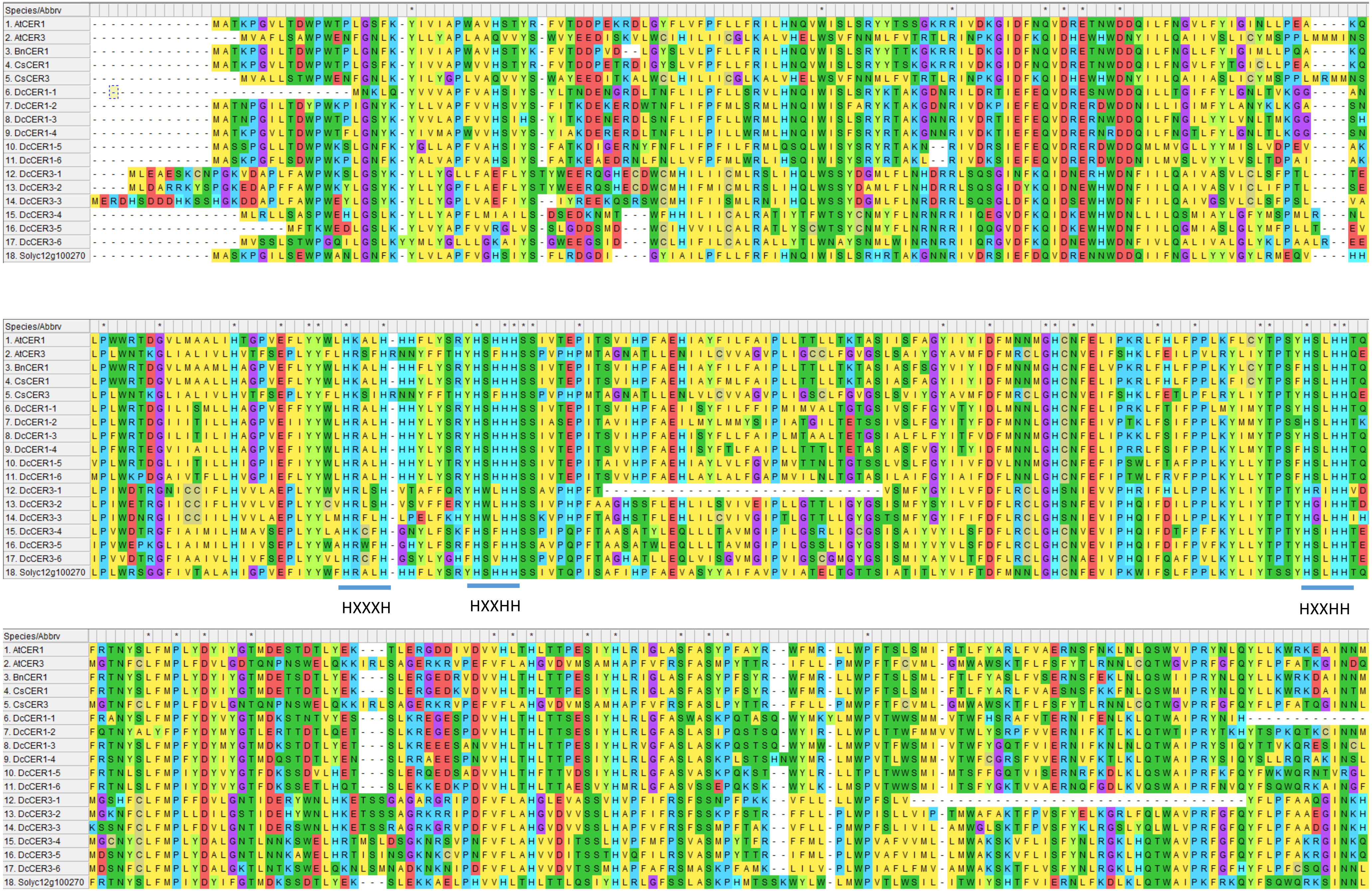
Species/Abbrv
1. AtCER1
2. AtCER3
3. BnCER1
4. CsCER1
5. CsCER3
6. DcCER1-1
7. DcCER1-2
8. DcCER1-3
9. DcCER1-4
10. DcCER1-5
11. DcCER1-6
12. DcCER3-1
13. DcCER3-2
14. DcCER3-3
15. DcCER3-4
16. DcCER3-5
17. DcCER3-6
18. Solyc12g100270
HXXXH
HXXHH
HXXHH

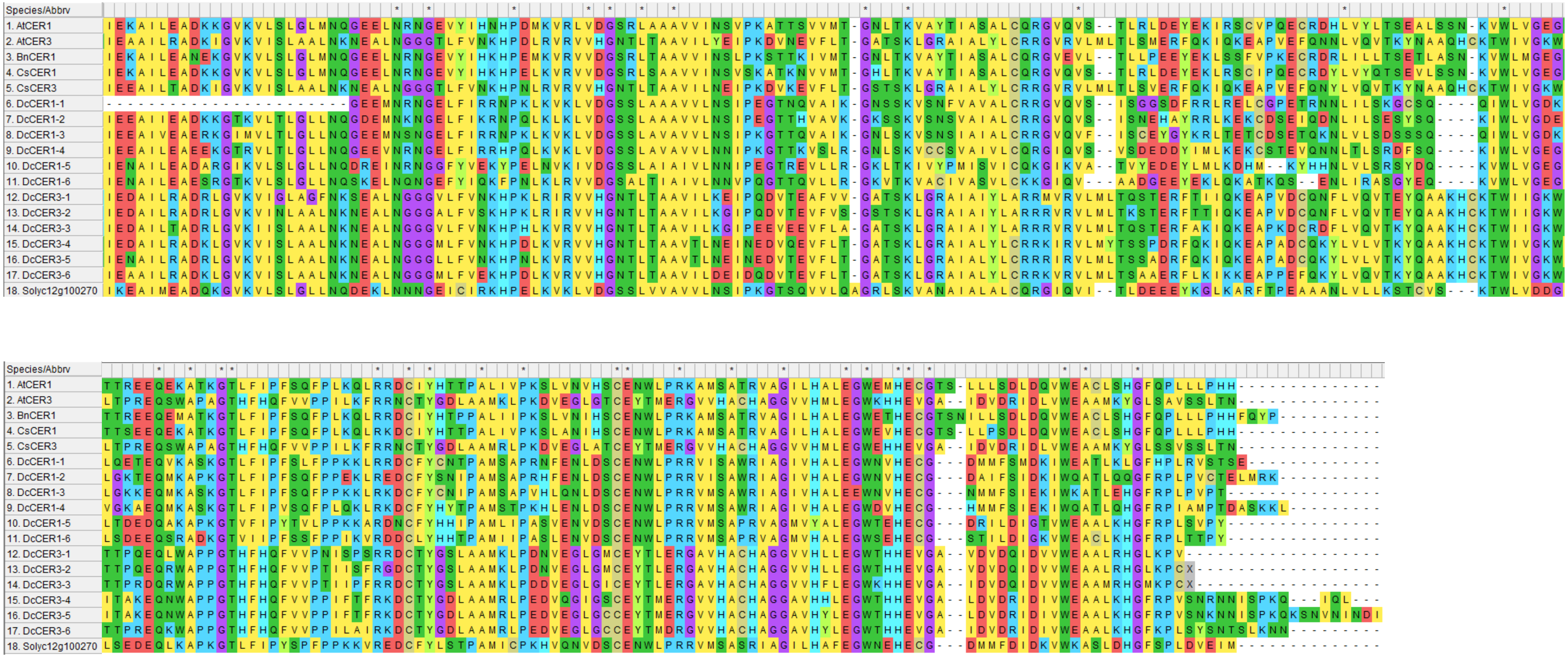

Figure S6 Alignment (MEGA-X, Muscle) of the predicted protein sequences of 12 *D. carota CER1* and *CER3* genes (see Table 2**)** and comparison with other known plant *CER1/3s* (*AtCER1*_NP_171723.2 and *AtCER3*_ NP_200588.2 from *A. thaliana*; *CsCER1*_KJ461885 and *CsCER3*_AIE57504.1 from *Camelina sativa; BnCER1*_KF724897 from *Brassica napus*) and the putative tomato *CER1* gene *Solyc12g100270* [27]. Three putative His boxes are underlined.
