# Supplementary material for: The genetic control of polyacetylenes involved in bitterness of carrots (Daucus carota L.): Identification of QTLs and candidate genes from the plant fatty acid metabolism
Source: BMC Plant Biol. 2022 Mar 2;22:92. doi: 10.1186/s12870-022-03484-1 (PMC8889737; doi:10.1186/s12870-022-03484-1)
Supplement: Supplementary file 10 — Additional file 10: Figure S7. Amino acid identity of carrot CER1 and CER3 proteins. [file 12870_2022_3484_MOESM10_ESM.pdf]

**Figure S7** Amino acid identity (%) of 12 CER1/3s identified in this study

| | 1 | 2 | 3 | 4 | 5 | 6 | 7 | 8 | 9 | 10 | 11 | 12 | | |
|---|---|---|---|---|---|---|---|---|---|---|---|---|---|---|
| **1** | | 73.6 | 78.2 | 72.9 | 58.0 | 58.6 | 33.3 | 32.7 | 33.2 | 33.5 | 34.9 | 33.9 | **1** | DcCER1-1 |
| **2** | | | 74.7 | 71.4 | 59.2 | 58.6 | 34.6 | 34.6 | 33.4 | 34.7 | 35.6 | 35.9 | **2** | DcCER1-2 |
| **3** | | | | 79.4 | 61.3 | 59.8 | 32.6 | 34.1 | 32.4 | 34.3 | 35.6 | 33.8 | **3** | DcCER1-3 |
| **4** | | | | | 61.6 | 61.1 | 34.2 | 34.6 | 33.6 | 34.3 | 35.7 | 34.7 | **4** | DcCER1-4 |
| **5** | | | | | | 72.5 | 34.2 | 34.4 | 34.3 | 35.1 | 35.5 | 36.1 | **5** | DcCER1-5 |
| **6** | | | | | | | 35.2 | 34.8 | 35.7 | 36.5 | 37.3 | 35.8 | **6** | DcCER1-6 |
| **7** | | | | | | | | 85.3 | 79.2 | 61.2 | 62.1 | 63.2 | **7** | DcCER3-1 |
| **8** | | | | | | | | | 80.3 | 62.3 | 63.1 | 63.5 | **8** | DcCER3-2 |
| **9** | | | | | | | | | | 63.2 | 63.7 | 63.7 | **9** | DcCER3-3 |
| **10** | | | | | | | | | | | 85.5 | 72.1 | **10** | DcCER3-4 |
| **11** | | | | | | | | | | | | 73.6 | **11** | DcCER3-5 |
| **12** | | | | | | | | | | | | | **12** | DcCER3-6 |
| | **1** | **2** | **3** | **4** | **5** | **6** | **7** | **8** | **9** | **10** | **11** | **12** | | |
