# Supplementary material for: The genetic control of polyacetylenes involved in bitterness of carrots (Daucus carota L.): Identification of QTLs and candidate genes from the plant fatty acid metabolism
Source: BMC Plant Biol. 2022 Mar 2;22:92. doi: 10.1186/s12870-022-03484-1 (PMC8889737; doi:10.1186/s12870-022-03484-1)
Supplement: Supplementary file 11 — Additional file 11: Figure S8. PA contents of cultivars AN, BRL and AN. [file 12870_2022_3484_MOESM11_ESM.pdf]

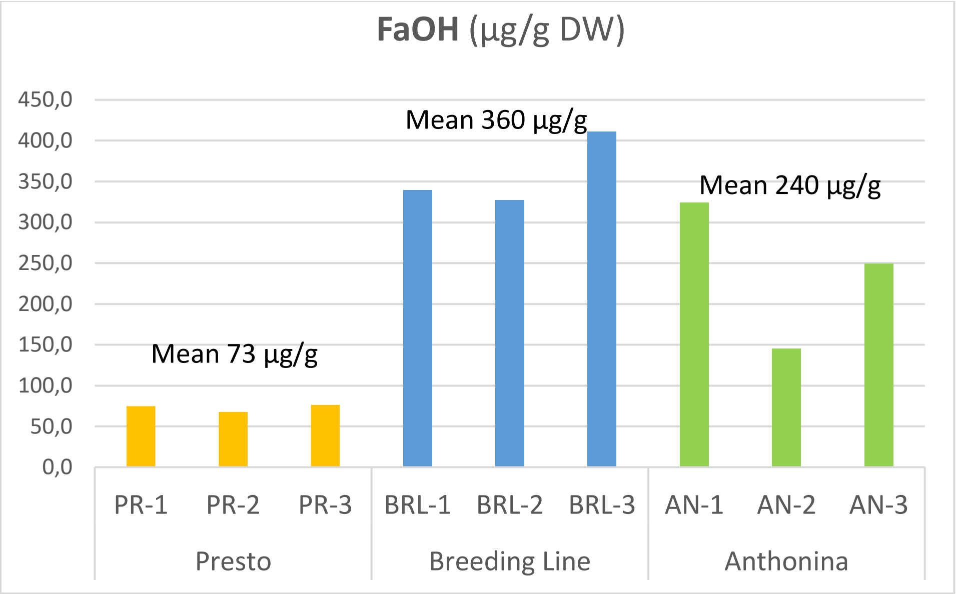

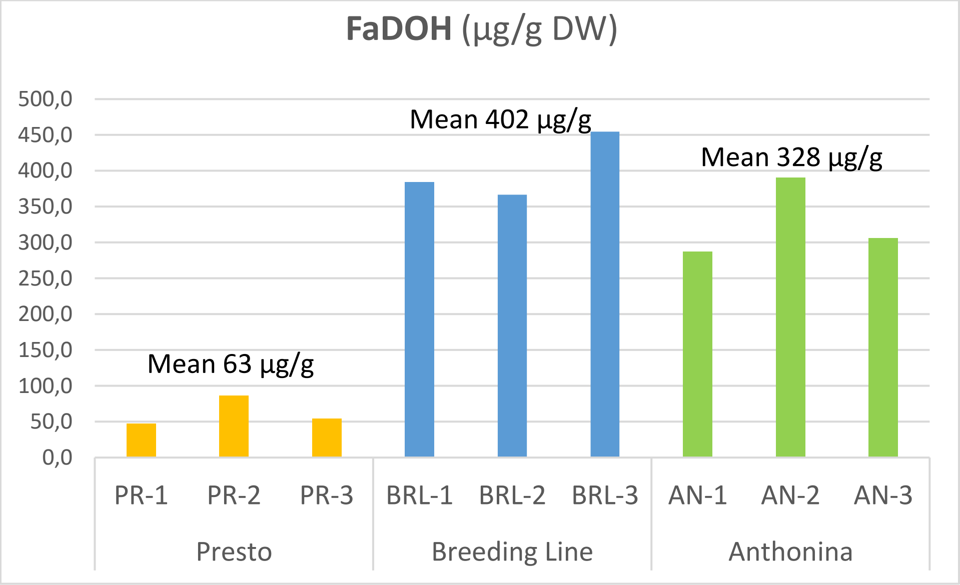

Figure S8 FaOH and FaDOH levels of each three individuals of cvs. 'Presto' (PR), 'Breeding line' (BRL) and 'Anthonina' (AN) based on analysis of each three root plugs (8 mm x 3 mm)
