# Supplementary material for: The genetic control of polyacetylenes involved in bitterness of carrots (Daucus carota L.): Identification of QTLs and candidate genes from the plant fatty acid metabolism
Source: BMC Plant Biol. 2022 Mar 2;22:92. doi: 10.1186/s12870-022-03484-1 (PMC8889737; doi:10.1186/s12870-022-03484-1)
Supplement: Supplementary file 12 — Additional file 12: Figure S9. Tissue-specific qRT-PCR of additional FAD2 genes in 3 cultivars. [file 12870_2022_3484_MOESM12_ESM.pdf]

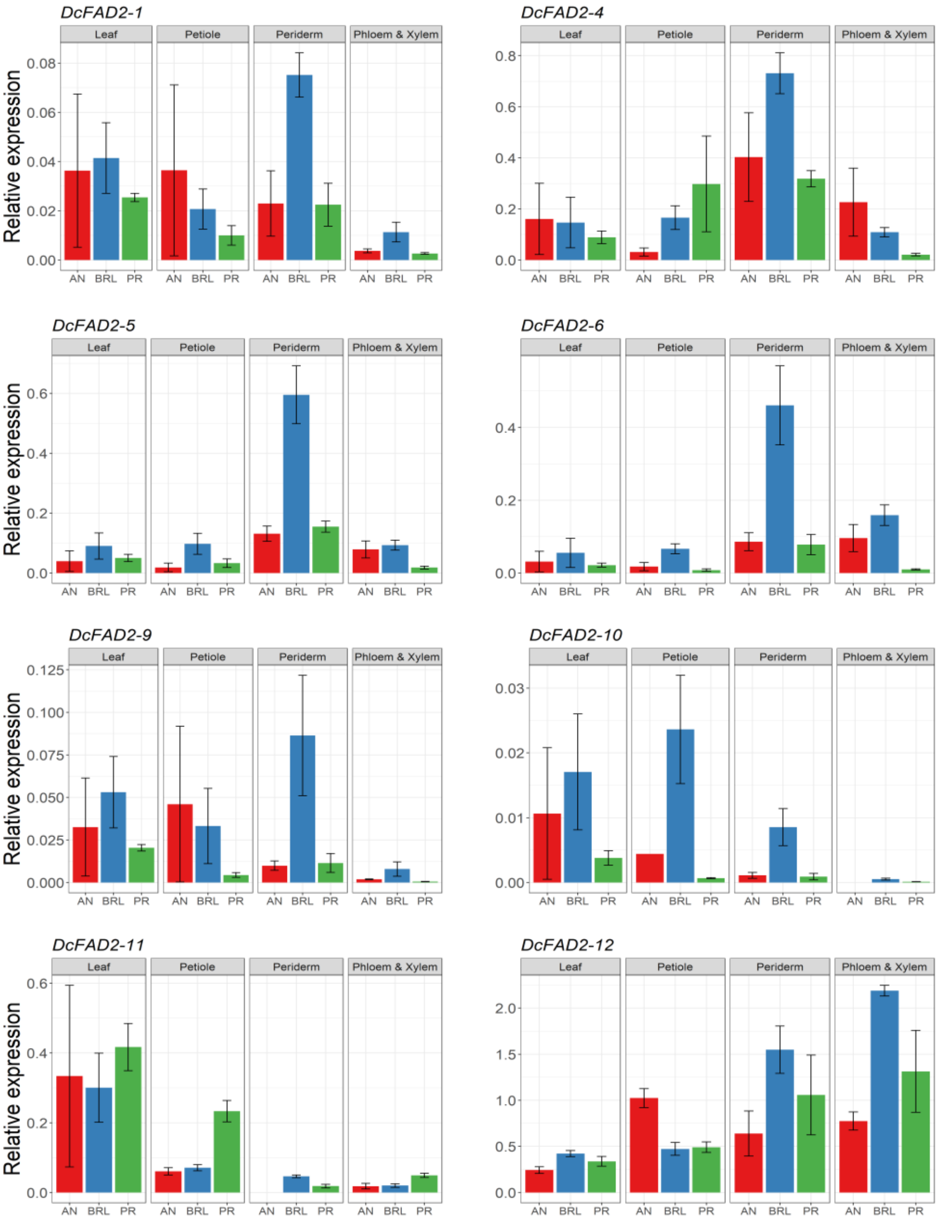

Figure S9 Tissue-specific expression profiles of additional *FAD2* genes associated with minor QTLs (*DcFAD2-1*, *DcFAD2-4, DcFAD2-6, DcFAD2-9, DcFAD2-11*) or not associated with QTLs (*DcFAD2-5, DcFAD2-10, DcFAD2-12*). The levels of RNA transcripts were analysed in leaf, petiole, periderm, and a mixture of phloem and xylem of Anthonina (AN), Breeding line (BRL) and Presto (PR). Data represent means of three individuals of each cultivar with error bars indicating standard error.
