# Supplementary material for: The genetic control of polyacetylenes involved in bitterness of carrots (Daucus carota L.): Identification of QTLs and candidate genes from the plant fatty acid metabolism
Source: BMC Plant Biol. 2022 Mar 2;22:92. doi: 10.1186/s12870-022-03484-1 (PMC8889737; doi:10.1186/s12870-022-03484-1)
Supplement: Supplementary file 13 — Additional file 13: Figure S10. qRT-PCR of candidate genes associated with major QTL in parents. [file 12870_2022_3484_MOESM13_ESM.pdf]

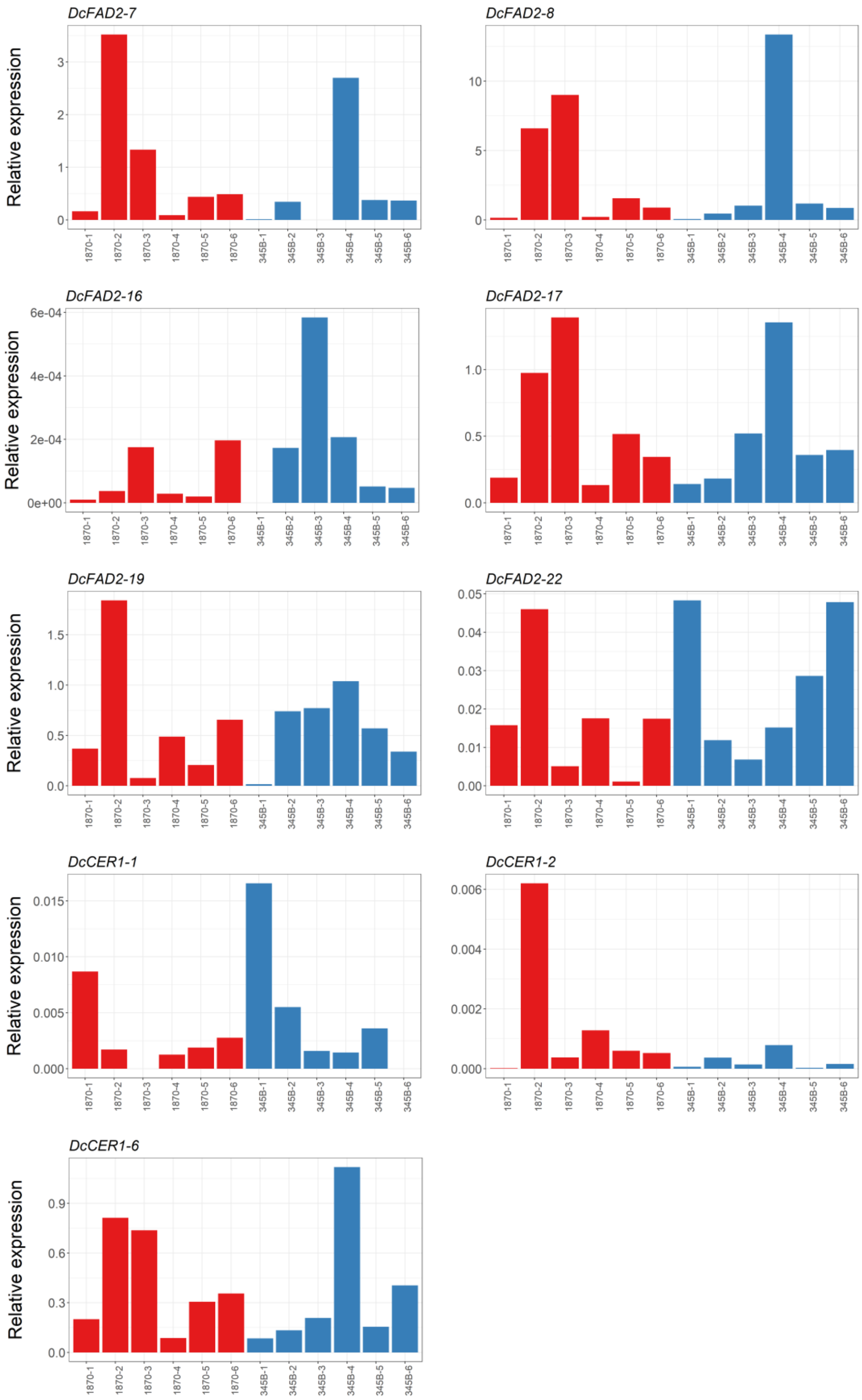

Figure S10 Expression profiles of six *DcFAD2* genes and three *DcCER1* genes located within 2-LOD QTL intervals on chromosomes 4 and 9, respectively. The levels of RNA transcripts for *DcFAD2-7*, *DcFAD2-8*, *DcFAD2-16*, *DcFAD2-17*, *DcFAD2-19*, *DcFAD2-22*, *DcCER1-1*, *DcCER1-2* and *DcCER1-6* were analysed in a mixed root sample (PPX method, see Mat. + Methods) from individual roots of six genotypes each of parental lines 1870 (red) and 345B (blue).
